# Medical device-based neuromodulation for motor symptoms in Parkinson’s disease: a systematic review and meta-analysis

**DOI:** 10.3389/fneur.2026.1731885

**Published:** 2026-02-12

**Authors:** Paulo E. P. Teixeira, João Victor Ribeiro, João Pedro Serrao Perin, Walter Augusto Fabris Moraes, Elly Angelica Pichardo, Ciro Ramos-Estebanez, Felipe Fregni, Tim Wagner, Laura Dipietro

**Affiliations:** 1 Highland Instruments, Cambridge, MA, United States; 2 Neuromodulation Center and Center for Clinical Research Learning, Spaulding Rehabilitation Hospital and Mass General Brigham, Cambridge, MA, United States; 3 São Leopoldo Mandic School of Medicine and Dentistry, Campinas, Brazil; 4 Universidade de São Paulo, São Paulo, Brazil; 5 Epidemiology Department, Harvard T. H. Chan School of Public Health, Boston, MA, United States; 6 Departments of Neurology and Rehabilitation, and Neurosurgery, University of Illinois Chicago, Chicago, IL, United States; 7 Physical Medicine & Rehabilitation Department, Harvard Medical School, Boston, MA, United States; 8 Harvard-MIT Division of Health Sciences and Technology, Cambridge, MA, United States

**Keywords:** brain stimulation, motor function, neuromodulation, Parkinson’s disease, systematic review and meta-analysis

## Abstract

**Objective:**

To synthesize randomized clinical trial (RCT) evidence comparing the efficacy of medical device-based neuromodulation (MDBN) techniques for improving motor symptoms in Parkinson’s disease (PD).

**Background:**

Device-based neuromodulation therapies, including deep brain stimulation (DBS), transcranial magnetic stimulation (TMS), and transcranial direct current stimulation (tDCS), are used to target PD motor symptoms, but reported effect sizes vary substantially across trials.

**Methods:**

A systematic review was conducted in accordance with PRISMA guidelines. RCTs evaluating MDBN for PD motor function, measured by the Unified Parkinson’s Disease Rating Scale Part III (UPDRS3), were identified through MEDLINE, Embase, and Web of Science. A random-effects meta-analysis was performed for the primary outcome (UPDRS3). Risk of bias was assessed using the Cochrane RoB tool, and certainty of evidence with GRADE. Meta-regression examined methodological, population, and intervention variables associated with effect size variation.

**Results:**

Forty-seven RCTs (56 intervention comparisons; 2,638 participants) were included. Thirty trials investigated non-invasive neuromodulation (mainly rTMS and tDCS) and 17 invasive approaches (DBS). Meta-analysis of 39 effect sizes (from studies during L-Dopa ON, near-term follow-up) showed a small but significant improvement with active MDBN (SMD = −0.18, *p* = 0.008; *I*^2^ = 61%). Meta-regression showed larger effects in studies using intention-to-treat analysis and smaller effects when sham stimulation was used as a comparator.

**Conclusion:**

MDBN is associated with modest yet significant motor improvements in PD. Effect size estimates are influenced by methodological characteristics, particularly analysis strategy and comparator selection. This is the first review in the field to calculate and incorporate separate effect sizes for each active intervention group in multi-arm trials, enabling more precise data inclusion.

## Introduction

Parkinson’s disease (PD) is a progressive, neurodegenerative condition (1) and the second most prevalent neurodegenerative disorder after Alzheimer’s disease, affecting 572 per 100,000 people aged 45 and older in the USA (2). Its characteristic features are motor symptoms – particularly bradykinesia, resting tremor, rigidity, and postural instability – with the latter typically appearing in later stages alongside non-motor symptoms such as sleep disturbance, depression, and cognitive decline (3). Dopamine is a key neurotransmitter that modulates motor control by fine-tuning basal ganglia circuitry, and its depletion leads to the characteristic motor symptoms of the disease (4). Standard pharmacological therapy aims to restore dopaminergic balance within basal ganglia circuits (5). Mainstay treatments include levodopa (L-dopa), dopamine agonists, Monoamine Oxidase-B (MAO-B) inhibitors, and Catechol-O-Methyltransferase (COMT) inhibitors, each acting through different mechanisms to enhance or mimic dopamine signaling (6), but providing only temporary symptomatic motor relief without halting the neurodegenerative process of PD. Over time, side effects of these medications include motor complications such as motor fluctuations and levodopa-induced dyskinesias. In addition, a decreased efficacy of these medications is seen over time (7).

Device-based neuromodulation therapies, such as deep brain stimulation (DBS) (8, 9), an established intervention, and other evolving approaches such as transcranial magnetic stimulation (TMS) (10) and transcranial direct current stimulation (tDCS) (11), are being used or investigated as treatment options for PD motor symptoms. These interventions offer targeted symptom relief and may improve motor function (12), though their effects on motor symptoms can vary (13). As the field continues to grow, accumulating evidence from multiple clinical trials, it is increasingly valuable to synthesize these findings through systematic reviews and meta-analyses.

To date, few systematic reviews have compared the effectiveness of different device-based neuromodulation therapies in PD (14, 15). Although these have provided valuable overviews, they are limited as they lack quantitative synthesis of outcomes (meta-analysis), subgroup analysis, and direct comparisons across intervention types. Quantitative analysis such as meta-analysis would provide pooled effect sizes, allowing for direct comparisons of treatment efficacy across devices and protocols while accounting for between-study heterogeneity. Additional quantitative analysis such as subgroup/meta-regression analyses would also allow for comparison of different categories of neuromodulation devices (e.g., invasive vs. non-invasive). Previous systematic reviews and/or meta-analytic studies have examined device-based neuromodulation for motor symptoms in PD, including those focusing on DBS and others on repetitive transcranial magnetic stimulation (rTMS) (16, 17). For example, a recent meta-analytic study found that rTMS produced significant motor improvements, particularly when high-frequency stimulation was applied over the primary motor cortex, with multi-session protocols yielding more sustained benefits (17). Similarly, multiple systematic reviews (18–20) have synthesized evidence supporting DBS as an effective intervention for motor symptom relief in PD. However, these reviews have largely been device-specific, and to our knowledge, no meta-analysis has comprehensively compared the relative effectiveness of the full range of available device-based interventions, both invasive and non-invasive, on motor symptoms, a core feature of PD. Given the growing body of RCTs involving diverse non-invasive and invasive neuromodulation devices, a comprehensive systematic review and meta-analysis is warranted.

The current study aims to systematically synthesize data from multiple RCTs to evaluate and compare the efficacy of different medical device-based neuromodulation (MDBN) techniques for improving motor symptoms in patients with PD. Such a study will allow quantification of treatment effects on motor outcomes across techniques, and also enable comparisons across device types, ultimately informing clinical decision-making in the management of PD motor symptoms.

## Methods

A systematic review and meta-analysis of RCTs was conducted to evaluate the effectiveness of various MDBN approaches in improving motor function among individuals with PD. To further investigate the variability in effect sizes, a meta-regression analysis was performed, examining both continuous and categorical factors related to study design, patient characteristics, and intervention parameters. The review adhered to the Preferred Reporting Items for Systematic Reviews and Meta-Analyses (PRISMA) guidelines (21).

### Search strategy

A comprehensive literature search was conducted across three major databases: MEDLINE, EMBASE, and Web of Science. A broad and inclusive search strategy was implemented (details in Appendix 1). The search incorporated various synonyms and related terms for PD, neuromodulation, and motor symptoms, including relevant Medical Subject Headings (MeSH). In addition, reference lists of key studies were reviewed (snowballing technique), and search completeness was verified through consultation with domain experts (TW, FF, LD). The search spanned from database inception through April 1, 2025, and was restricted to articles published in English. A clinical trial filter was used for all databases.

### Selection criteria

Research articles investigating the effects of any MDBN intervention in improving motor function in PD patients, as assessed by Unified Parkinson’s Disease Rating Scale Part III (UPDRS3), were included if they met the following criteria: (1) were RCTs – not including cross-over designs; (2) included only subjects diagnosed with PD; (3) studies that investigated one or more neuromodulation interventions (e.g., but not limited to, DBS, TMS, Vagus Nerve Stimulation (VNS), etc.) as a treatment for motor symptoms in PD; (4) studies that included a comparison group receiving either a placebo, a sham treatment, standard medical treatment, or another neuromodulation intervention; (5) measured motor symptoms as a primary or secondary outcome via UPDRS3; and (6) only peer-reviewed articles were considered. Journal articles were excluded if they: (1) were not RCTs (e.g., but not limited to, cohort studies, case–control studies, cross-sectional studies, observational studies); (2) involved patients without a confirmed diagnosis of PD or focused on specific subgroups inside PD (e.g., PD with aphasia, PD with depression, tremor dominant PD, etc.), or studies that focused on other neurological conditions; (3) did not evaluate neuromodulation interventions (e.g., but not limited to, pharmacological treatments, physical therapy, surgical interventions other than neuromodulation); (4) did not measure motor function using the UPDRS3; (5) provided incomplete or not sufficient data needed for meta-analysis (such as UPDRS3 central tendency and variability values for all groups); (6) were non-peer-reviewed articles, such as conference abstracts, letters to the editor, commentaries, and case reports.

### Article screening

A web-based platform, Covidence, was employed to manage and screen all records identified through the search. The screening process was conducted in two stages: initially, titles and abstracts were reviewed to identify potentially relevant studies. Articles deemed suitable at this stage proceeded to a full-text review, where inclusion and exclusion criteria were applied. Two reviewers (PEPT and WAFM), both trained in evidence synthesis and clinical trial methodology, independently screened all records. In cases of disagreement during eligibility assessment, both reviewers re-evaluated the full text to reach a consensus. If discrepancies remained unresolved, a senior reviewer (FF) conducted an independent assessment to make the final determination.

### Data extraction

Data extraction was carried out by three authors (PEPT, JPSP, and JVR), who independently populated a standardized spreadsheet designed to capture all predefined variables. Information related to study design, participant characteristics, and intervention details was systematically collected to support the creation of analytic variables used in subsequent analyses (see Table 1).

**Table 1:** Aspects of study design, participant characteristics, and intervention features used to derive the variables used for analysis.

Study methodology	Population characteristics	Intervention features
Number of intervention groups	Age	Type of medical device used
Use of intention-to-treat analysis	Duration of symptoms	Use of a combined intervention
Number of follow-up time points for UPDRS3 outcome	Gender	Type of combined intervention (e.g., exercise-based; another MDBN; motor-learning based)
Sample size (n) per intervention group	Race	Used sham group comparison
Timing of follow-ups (in months)	Hoehn & Yahr score	Invasive or non-invasive intervention
Conflict of interest reported	Baseline independence level	Frequency of intervention sessions per week (if applicable)
Use of MDS-UPDRS3 (instead of UPDRS3)	Baseline depression level	Duration of intervention (in weeks)
	Baseline UPDRS3 scores (ON and OFF states)	Patient setting (e.g., inpatient, outpatient)
	Percentage of subjects using L-dopa	Brain target (e.g., M1, cerebellum)
	L-dopa equivalent daily dose (mg)	

UPDRS3, Unified Parkinson’s Disease Rating Scale Part III; mg, Milligrams; M1, Primary motor cortex.

As part of the data extraction, central tendency and variability estimate scores of the UPDRS3 from all time points used in the studies were collected from the included studies to be used in the meta-analysis. Effect sizes were calculated from the UPDRS3 scores. In studies that included more than one active device-based intervention arm (e.g., two active treatments and one sham), separate effect sizes were calculated for each active–sham comparison. The web-based software WebPlotDigitizer (version 5.2) (22) was used to extract data when articles did not include specific values in tables or in text.

### Risk of bias assessment

The risk of bias was assessed using the RoB-2 (Revised Cochrane Risk of Bias Tool) (23), that evaluates the following domains: (1) randomization process, (2) deviations from intended interventions, (3) missing outcome data, (4) measurement of the outcome, (5) selection of the reported result – each analyzed through structured subdomains. All data were systematically organized using Microsoft Excel. Two independent reviewers (JPSP and JVR), both with training in clinical research,^1^ performed the assessments. Discrepancies were resolved through discussion, with emphasis on understanding the underlying reasons for divergence. Overall bias was categorized as low risk, some concerns, and high risk. Assessors rated bias independently and crosschecked results with one another. Any conflicting results were discussed with a senior author to reach the final decision.

### Evidence certainty and quality of evidence

Two reviewers (PEPT, EAP) independently assessed the certainty of evidence using the GRADE framework (21), which considers factors like study design, risk of bias, inconsistency, and precision. This process was used to rate the quality of evidence for the UPDRS3 outcome. A Summary of Findings table was built to summarize the results of this evaluation.

### Publication bias

To assess potential publication bias, funnel plot asymmetry was visually inspected, complemented by formal statistical tests including Egger’s test and Begg’s test, with continuity-corrected *p*-values used to determine significance. When evidence of bias was detected, the Duval and Tweedie (24) nonparametric “trim and fill” approach was applied to adjust for missing studies and generate a corrected estimate of the overall effect size (ES).

### Statistical analysis

#### Main effect

Analysis of the comparison of the efficacy of different MDBN interventions for improving motor symptoms in patients with PD was reported as a pooled effect of all selected trials. A random-effects model was used with weights based on the DerSimonian and Laird method (25) using standardized mean differences—SMD (Hedges’ g), as it was expected that the included trials assessed motor function using different versions of the UPDRS3 (MDS-UPDRS vs. UPDRS) outcome measure. Results were reported in a forest plot using the SMD along with their 95% confidence intervals. For clinical interpretability, results of the primary analysis were converted to UPDRS3 units by back-transformation method using a representative reference standard deviation (26–28). For trials evaluating multiple active device-based interventions, effect sizes were calculated separately for each intervention arm and adjusted using a correlation coefficient of 0.55 to account for the non-independence of comparisons. This value was selected based on prior literature (29), as correlation data for UPDRS3 scores at baseline were not available in the original articles. To test the robustness of this assumption, a sensitivity analysis was conducted using alternative correlation values of 0.25, 0.50, and 0.75. Statistical heterogeneity across studies was assessed using the *I*^2^ statistic and Cochran’s Q test, with an *I*^2^ value above 50% indicating moderate to high heterogeneity (30). An additional forest plot illustrating the weighted percentage improvement in UPDRS3 scores was created. This analysis was included to provide a more intuitive measure of clinical benefit, allowing for direct comparison of effect sizes across studies regardless of baseline UPDRS3 scores, and to help readers better interpret the clinical relevance of the findings in the context of PD management. Descriptive statistics were used to summarize key characteristics of the included studies, including methodological features, population demographics, and intervention details.

### Sensitivity analysis

To account for the expected variation in the timing of UPDRS3 outcome assessments across studies, a sensitivity analysis was planned *a priori* for near, short, and long-term follow-up data. Follow-up periods were categorized as near-term (<4 months), short-term (4–12 months), and long-term (>12 months) based on when post-intervention outcome data were collected. If multiple outcome time points were available within a follow-up category, the time point closest to a reference mark was selected: near-term (<4 months) used the time point nearest to 4 months, short-term (4–12 months) used the point closest to 8 months (the midpoint), and long-term (>12 months) used the time point closest to 12 months. Additional pre-specified sensitivity analyses included stratifying results by whether outcomes were assessed during the ON or OFF state of L-dopa equivalent medication regardless of the duration post-intervention (using the latest available follow-up data from each study), comparing invasive versus non-invasive neuromodulation interventions, studies that used the MDS-UPDRS instead of the traditional UPDRS to measure motor symptoms, and excluding studies that fell outside the expected range in the funnel plot for publication bias.

### Meta-regression

Study variables related to methodology, population, and intervention were analyzed to explore potential sources of effect size heterogeneity. Meta-regression was performed for both continuous and categorical variables.

To minimize type I error associated with multiple testing (31), variable selection followed a structured model-building process (32). Univariate meta-regressions were first performed to identify potential covariates, using a *p*-value threshold of <0.20 (32) to retain variables for further analysis. This step reduced the number of variables considered in multivariate modeling. Significant variables were then included in an initial multivariate model, and a backward elimination approach was applied by sequentially removing the least significant variables until only predictors with *p*-values <0.05 remained.

For all meta-regression models, linearity was evaluated through scatterplot inspection with a superimposed regression line. Homoscedasticity was assessed by plotting standardized predicted values against standardized residuals (33). Residual normality was tested using the Shapiro–Wilk test (34). Given the exploratory nature of the analysis, no adjustment for multiple testing was applied. All tests were two-tailed with a significance level set at *p* < 0.05, and analyses were conducted using Stata V17 BE (StataCorp, College Station, TX).

## Results

**Figure 1:**
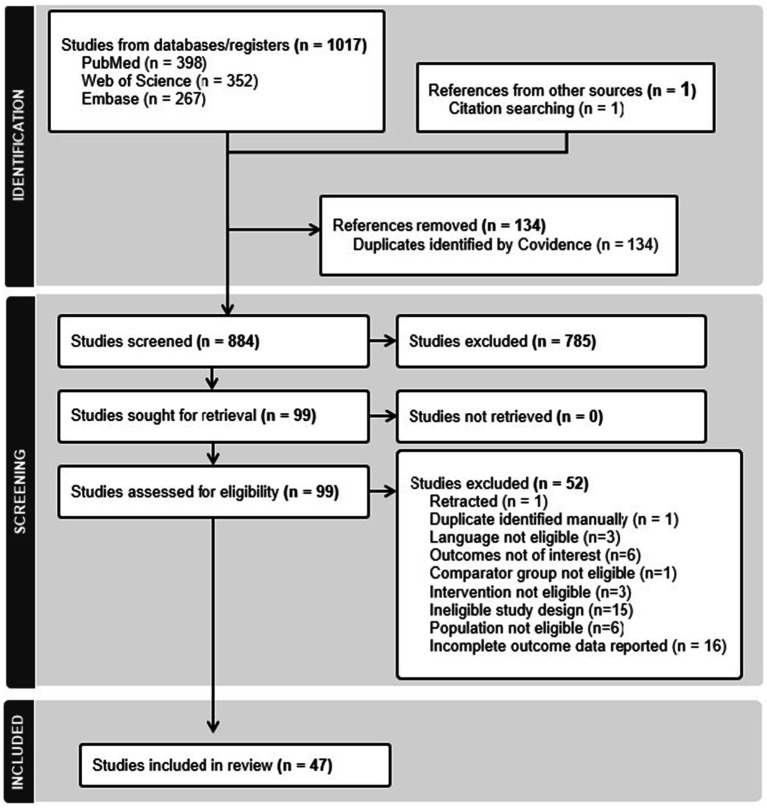
Flow diagram of study selection.

A total of 1,017 articles were initially identified through the primary search strategy, with one additional study found via snowballing. Following the removal of duplicates, 883 articles proceeded to the initial screening stage. After completing all screening phases, 47 studies met the inclusion criteria. The selection process is illustrated in the CONSORT diagram (Figure 1).

Of the 47 studies included in the review, several trials featured multiple intervention arms, with either more than one active device group compared to a shared sham control, or multiple active device interventions compared to each other. In such cases, separate effect sizes were calculated for each active arm relative to the designated comparator (sham or specified reference intervention). The included studies were conducted between 1999 and 2024 across 18 countries, with the majority conducted in the USA (35–45) (*n* = 11) and China (10, 46–55) (*n* = 10). Additional contributions came from Spain (56–58) (*n* = 3), Italy (59, 60) (2), Germany (61–63) (3), Japan (64, 65) (2), and the Netherlands (66–68) (3), Hungary (69, 70) (2), South Korea (71, 72) (2), Argentina (73) (1), Turkey (74) (1), Egypt (75) (1), France (76) (1), Switzerland (46) (1), Israel (77) (1), Sweden (78) (1), Russia (79) (1), and Brazil (80) (1). Across all studies, a total of 2,638 participants were included. The mean age was 62.46 years, the average Hoehn & Yahr stage was 2.43, and the average symptom duration was 7.35 years. Motor outcomes were primarily assessed using the UPDRS3 scale (*n* = 40), while the MDS-UPDRS scale was used in 7 studies. In terms of intervention type, 30 studies examined non-invasive neuromodulation techniques – such as rTMS and tDCS – whereas 17 studies evaluated invasive methods (DBS). Regarding intervention design, most studies (*n* = 39) tested a non-combined intervention. Eight studies employed combined interventions, including the combination of either a device, exercise, motor-learning, or cognitive-based approach (Supplementary Table 1).

### Main effect

For the main analysis, we focused on the near-term follow-up used in each study and conducted in the L-dopa ON state because the majority of included studies reported outcomes under this condition. Other subgroups were explored (see details on “Sensitivity analysis” section).

**Figure 2:**
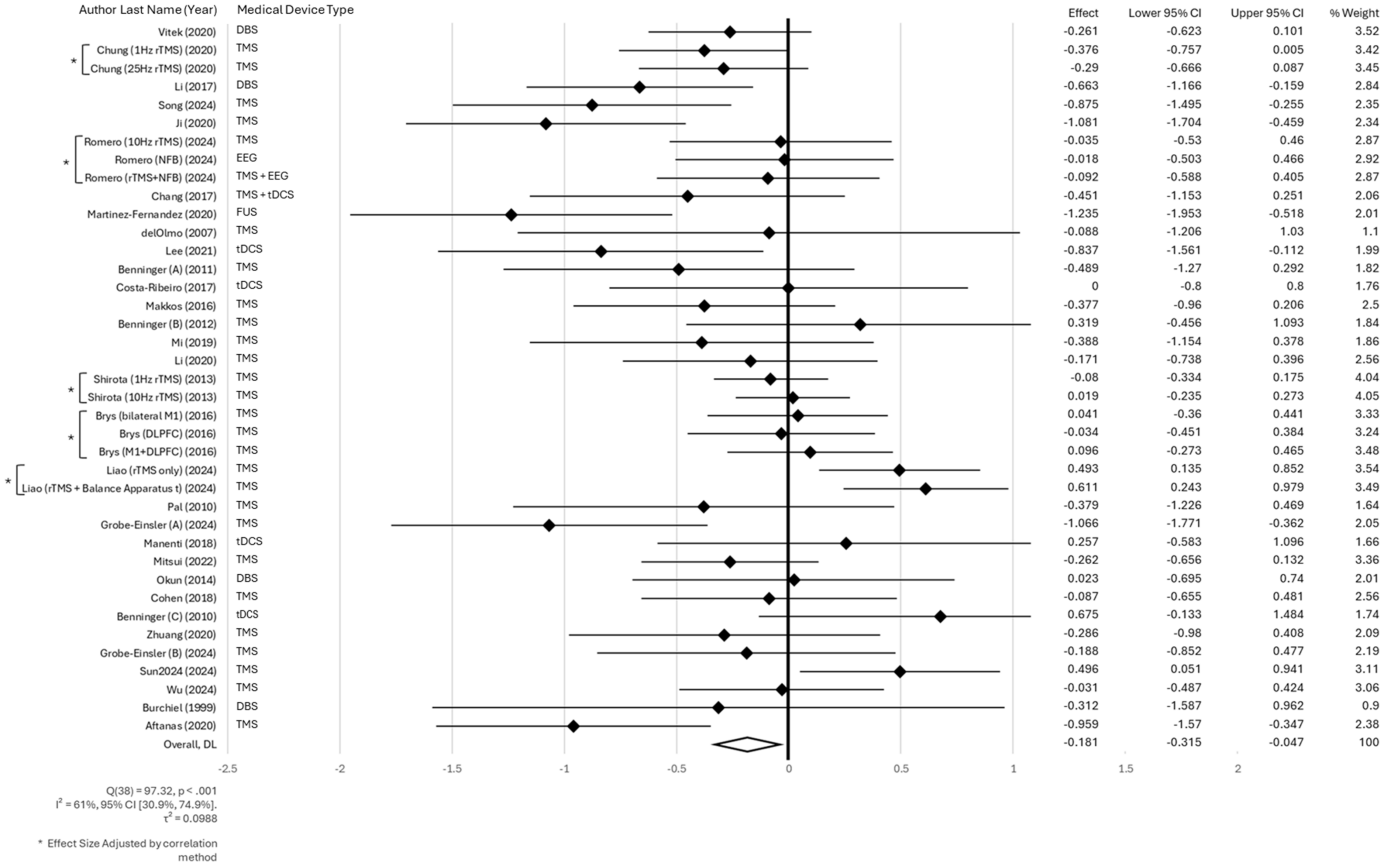
Forest plot for the main effect of different MDBN interventions for improving motor symptoms in patients with PD.

The meta-analysis assessing the efficacy of different MDBN interventions for improving motor symptoms in patients with PD demonstrated a significant benefit in favor of active MDBN interventions. A random-effects meta-analysis was conducted across 39 effect sizes. The overall effect was statistically significant, *z* = −2.64, *p* = 0.008, with a pooled effect size of −0.18, 95% CI [−0.31, −0.05] (where the reduction indicates an improvement). Heterogeneity was moderate to high, Q(38) = 97.32, *p* < 0.001, *I*^2^ = 61, 95% CI [30.9, 74.9%]. The between-study variance was τ^2^ = 0.0988. The H statistic was 1.60, 95% CI [1.20, 2.00], indicating that variability across studies exceeded sampling error. This represents a small but statistically significant effect size according to established benchmarks (81). Sensitivity analyses using correlation values of 0.25, 0.50, and 0.75 showed no meaningful impact on the results. Therefore, we used a correlation coefficient of 0.55 in the primary analysis, consistent with prior literature (29, 82), to adjust for non-independence in trials with multiple intervention arms. The forest plot for these findings is presented in Figure 2.

**Figure 3:**
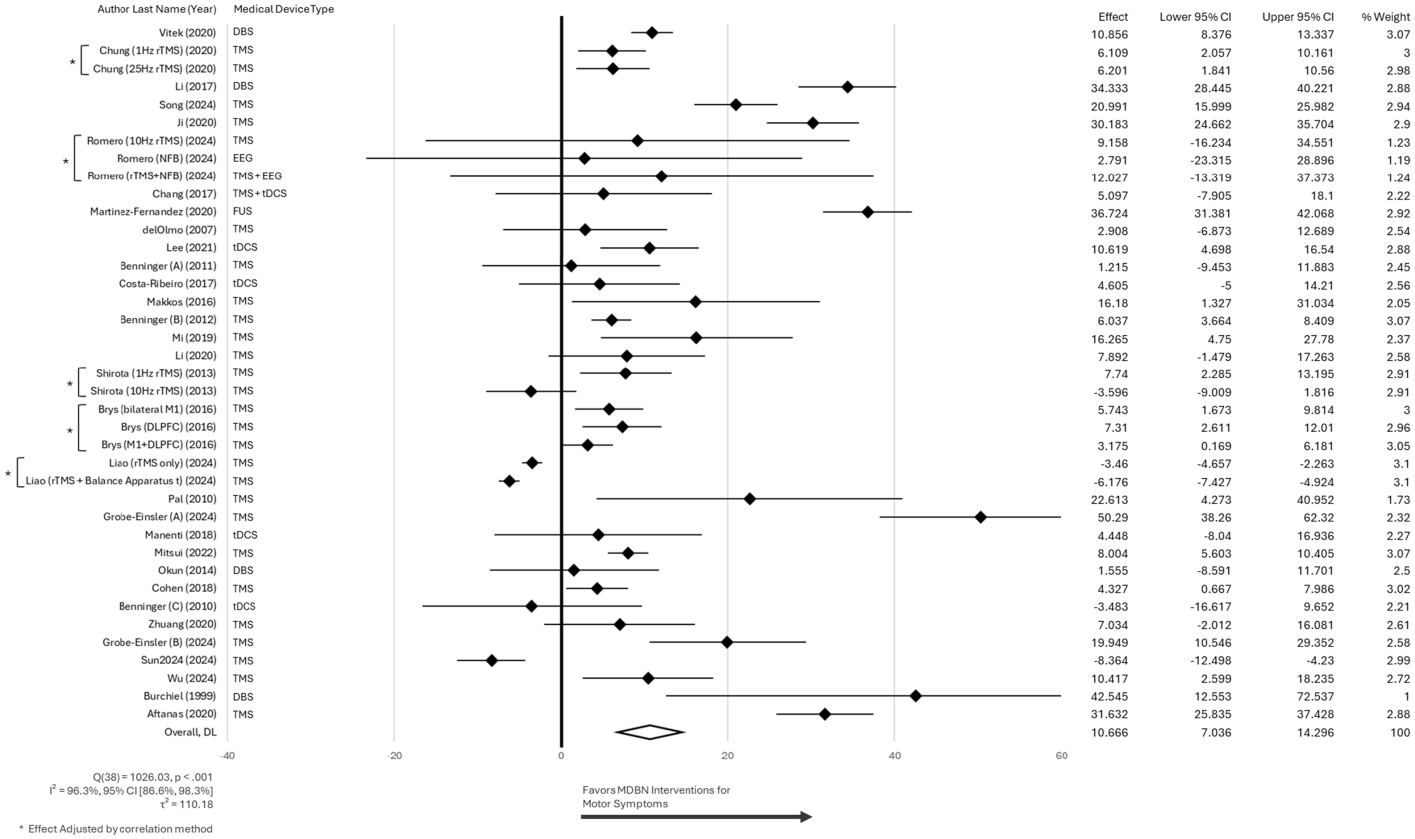
Forest plot for the main effect of different MDBN interventions for improving motor symptoms in patients with PD using the weighted percentage improvement from baseline of the UPDRS3.

Analysis of the pooled data from 39 studies assessing the percentage improvement in UPDRS3 scores with MDBN interventions in PD revealed a significant overall benefit for active treatment. The random-effects model produced a pooled estimate of 10.67% improvement (95% CI [7.04, 14.30]), and the overall effect was statistically significant (*z* = 5.76, *p* < 0.001). The analysis demonstrated considerable high heterogeneity, with Cochran’s Q(38) = 1026.03, *p* < 0.001, and *I*^2^ = 96.3% (95% CI [86.6%, 98.3%]), indicating substantial variability across studies. The estimated between-study variance (τ^2^) was 110.18, and the H statistic was 5.20 (95% CI [2.73, 7.68]). The high heterogeneity observed in the percentage improvement analysis reflects pronounced between-study variability and does not represent a consistent treatment effect, warranting caution in interpretation. The forest plot for these findings is presented in Figure 3.

### Meta-regression

Given the moderate to high heterogeneity observed in the main effect analysis (*I*^2^ = 61%), a meta-regression was conducted to explore potential sources of variability in effect sizes across studies. The meta-regression analysis was restricted to studies with near-term follow-up assessed in the L-dopa ON state, as the majority of all included studies reported outcomes under these conditions. The final meta-regression model included 35 studies and two study-level characteristics: use of intention-to-treat (ITT) analysis and sham group comparison. All other variables were not significant and consequently not included in the final model. The meta-regression model accounted for a substantial portion of the between-study variance (Adjusted R^2^ = 65.88%; *F*(2,33) = 11.06; *p* = 0.0002). Studies utilizing ITT analysis demonstrated significantly larger treatment effects (MD = 0.62; 95% CI: 0.29 to 0.94; *p* = 0.001) as compared to studies that did not use ITT analysis. In contrast, studies with a sham comparator group showed significantly smaller effects compared to those without sham (MD = −0.29; 95% CI: −0.55 to −0.03; *p* = 0.028).

### Risk of bias assessment

The majority of studies demonstrated low risk across all five assessed domains, including the randomization process, deviations from intended interventions, missing outcome data, outcome measurement, and selection of the reported result. Two studies (56, 61) were rated as high risk in the randomization process (D1 – Bias arising from the randomization process). Six studies were rated as having some concerns in at least one domain, with most common issues found in outcome measurement (D4 – Bias in measurement of the outcome). Overall, most studies were rated as having a low risk of bias. We provide the risk of bias assessment of the included RCTs in Appendix 1.

### Evidence certainty

The GRADEpro GDT software (83) was used to rate the quality of the evidence for the UPDRS3 outcome at the last follow-up used in each study and only on studies in which the assessments were conducted in the L-dopa ON state. The certainty of evidence was rated as “Low” due to downgrades for inconsistency and imprecision. With respect to risk of bias, two studies were judged to have a high risk in the randomization process and six had some concerns (primarily related to outcome measurement – D4). However, the majority of studies were rated as low risk of bias. Considering the overall distribution of risk across the body of evidence, and consistent with the GRADE guidance, no additional downgrade was applied for risk of bias. The evidence was downgraded one level due to inconsistency demonstrated by the presence of moderate heterogeneity. It was further downgraded due to imprecision, demonstrated by the predominance of small studies (fewer than 300 participants), with many underpowered, and because the 95% confidence interval indicated a small effect size. Additionally, evidence of small-study effects was detected by Egger’s test (*p* = 0.031). The trim-and-fill sensitivity analysis did not impute any additional studies, and the adjusted effect size remained unchanged (SMD = −0.116; 95% CI: −0.194 to −0.037), suggesting minimal impact of potential publication bias on the pooled effect estimate. Therefore, these findings do not indicate publication bias with a meaningful impact on the results, and downgrading the certainty of evidence under the publication bias domain is not warranted. A “Summary of findings” (Table 2) was created.

**Table 2:** The GRADE “Summary of findings” table for quality of evidence and magnitude of effect of the interventions.

Device-based neuromodulation for motor symptoms in Parkinson’s disease: a systematic review and meta-analysis
Patient or population: Patients with diagnosed PD
Settings: Outpatient
Intervention: Device-based neuromodulation with or without a combined intervention
Comparison: Sham or another active neuromodulation intervention

Outcomes	Effect size	Absolute effect (average score improvement (reduction) in UPDRS3 scores (95% CIs) in relation to baseline score)	No of participants (studies)	Quality of the evidence (GRADE)
UPDRS3 scores – Levodopa “ON” status during UPDRS scoring				
UPDRS3 scores (motor symptoms) improvement	SMD = −0.18 [−0.31 to −0.05]	This equates to a ≈ −2.16 points, 95% CI (−2.9 to −1.46) reduction in UPDRS3 raw scores	2,638 participants (47 studies)	⊕◯◯◯ Low ^1,2,4^
Comments: Downgraded for imprecision and inconsistency: Most studies had <300 participants; some studies had risk of bias in randomization (D1) or outcome measurement (D4).				
GRADE Working Group grades of evidence *High quality*: we are very confident that the true effect lies close to that of the estimate of the effect. *Moderate quality*: we are moderately confident in the effect estimate; the true effect is likely to be close to the estimate of effect, but there is a possibility that it is substantially different. *Low quality*: our confidence in the effect estimate is limited; the true effect may be substantially different from the estimate of the effect. *Very low quality*: we have very little confidence in the effect estimate; the true effect is likely to be substantially different from the estimate of effect.				

^1^Downgraded twice. Single randomized study (with under 300 participants) was considered inconsistent, imprecise (i.e., wide 95%CI), providing low quality of evidence.

^2^Downgraded once for inconsistency due to heterogeneity.

^3^Downgraded once for study limitation due to high or unclear risk of bias.

^4^Downgraded once for imprecision due to small sample size.

MD, mean difference; CI, confidence interval.

### Publication bias

The funnel plot (Appendix 1) displayed an overall symmetric distribution of study-level effects, with most points closely aligned within the funnel boundaries. However, minor asymmetry may be visually apparent, particularly among studies with larger standard errors. Egger’s test was conducted to formally assess small-study effects, and the test indicated statistically significant evidence of such effects (bias = −1.53; 95% CI: −2.92 to −0.15; *p* = 0.031), suggesting the possibility of publication bias or inflated effects in smaller studies. To further evaluate the potential impact of publication bias, a nonparametric trim-and-fill analysis was performed. The analysis did not impute any additional studies, and the adjusted pooled effect size (−0.116; 95% CI: −0.194 to −0.037) remained unchanged compared to the original estimate. While Egger’s test indicated small-study effects, the absence of imputed studies in the trim-and-fill analysis and the unchanged pooled SMD suggest that any potential publication bias is unlikely to meaningfully alter the results. The publication bias analysis was restricted to the same subgroup as the main meta-analysis, focusing on studies with near-term follow-up conducted in the L-dopa ON state.

### Sensitivity analysis

#### Follow-up duration

As defined *a priori*, a sensitivity analysis was conducted to examine differential effects of neuromodulation across three timeframes: near-term (<4 months), short-term (4–12 months), and long-term follow-up (>12 months). This analysis was limited to the subgroup of studies conducted in the L-dopa ON state. Thirty-nine comparisons from 32 studies contributed to the near-term analysis, yielding a pooled effect size (ES) of −0.181 [95% CI: −0.315 to −0.047; *p* = 0.008], with substantial heterogeneity observed (*I*^2^ = 61.0%; Cochran’s Q(38) = 97.32, *p* < 0.001; τ^2^ = 0.099). In the short-term subgroup, 12 comparisons from 11 studies were included, and the pooled ES was −0.135 [95% CI: −0.244 to −0.027; *p* = 0.014], with no significant heterogeneity detected (*I*^2^ = 0.0%; Cochran’s Q(11) = 4.90, *p* = 0.936; τ^2^ = 0.000). Finally, 10 studies contributed to the long-term analysis (>12 months), resulting in a non-significant pooled ES of −0.079 [95% CI: −0.250 to 0.092; *p* = 0.366], with no significant heterogeneity observed (*I*^2^ = 0.0%; Cochran’s Q(9) = 5.40, *p* = 0.798; τ^2^ = 0.000). Overall, these results suggest that small and statistically significant effects may be present in the near- and short-term, but such benefits appear to diminish over time and are not sustained at long-term follow-up (forest plots for all sensitivity analyses are detailed in Appendix 1). Conclusions regarding long-term efficacy are largely derived from studies of invasive DBS. In contrast, the long-term effects of non-invasive neuromodulation (e.g., rTMS or tDCS) remain unclear due to the lack of studies with extended follow-up.

### Medication state at outcome assessment

A sensitivity analysis was conducted by stratifying studies based on whether the UPDRS3 assessment was performed in the ON or OFF L-dopa state, using the most recent (last) follow-up data available from each study (regardless of follow-up duration). Among studies assessed in the ON state (k = 49), the pooled SMD favored active intervention over control (SMD = −0.150; 95% CI: −0.256 to −0.045; *p* = 0.005), with moderate heterogeneity (*I*^2^ = 52.0%). In contrast, studies assessed in the OFF state (*k* = 23) showed a slightly larger pooled effect (SMD = −0.292; 95% CI: −0.557 to −0.027; *p* = 0.031), though heterogeneity was substantial (*I*^2^ = 83.9%). These results indicate that therapeutic effects were present in both medication states, but findings in the OFF state were more heterogeneous across studies.

### Intervention type

A sensitivity analysis was conducted based on intervention type, comparing studies using invasive versus non-invasive neuromodulation modalities. Among studies utilizing invasive interventions (*n* = 14), the pooled SMD was −0.177 (95% CI: −0.297 to −0.057; *p* = 0.004), with no evidence of heterogeneity (*I*^2^ = 0.0%). In contrast, studies using non-invasive modalities (*n* = 28; including 35 intervention protocols) also demonstrated a significant effect in favor of active intervention (SMD = −0.163; 95% CI: −0.306 to −0.020; *p* = 0.025), though with moderate heterogeneity (*I*^2^ = 62.6%).

### Outcome measurement scale

A sensitivity analysis was conducted to examine whether the outcome measurement scale influenced treatment effect estimates. In studies that used the traditional UPDRS (*n* = 40), the pooled SMD was −0.091 (95% CI: −0.200 to 0.018; *p* = 0.101), with moderate heterogeneity (*I*^2^ = 47.7%). In contrast, studies that utilized the MDS-UPDRS (*n* = 7) demonstrated a significantly larger effect size (SMD = −0.483; 95% CI: −0.733 to −0.232; *p* < 0.001), with low-to-moderate heterogeneity (*I*^2^ = 34.2%). These results are inconsistent with the meta-regression analysis, which did not identify the use of the MDS-UPDRS as a significant moderator. In this sensitivity analysis, the use of the MDS-UPDRS was associated with greater treatment effects compared to the traditional UPDRS.

### Publication bias influence

Analysis of publication bias identified eight studies, representing nine intervention protocols, that fell outside the 95% confidence funnel limits: Song 2024, Ji 2020, Martinez-Fernandez 2020, Liao 2024 (both interventions), Grobe-Einsler 2024, Benninger 2010, Sun 2024, and Aftanas 2020. Sensitivity analysis excluding these studies included 30 comparisons and yielded a pooled SMD of −0.143 (95% CI: −0.231 to −0.056; *p* = 0.001), with no evidence of heterogeneity (*I*^2^ = 0.0%). These findings are consistent with the primary analysis, indicating that the overall results are robust to the exclusion of potential outliers or influential studies.

Additional exploratory subgroup analyses considering stimulation target and stimulation protocol for rTMS (high-frequency rTMS vs. low-frequency) are presented in Appendix 1. Subgroup analysis by tDCS stimulation polarity (anodal vs. cathodal) was not feasible because all included tDCS studies employed anodal stimulation.

## Discussion

The current study included 47 RCTs evaluating MDBN interventions against sham or active comparators. Such an analysis was warranted given the growth in MDBN trials over recent decades and the variability in reported effect sizes. To our knowledge, this is the first meta-analysis in the field that calculates and incorporates separate effect sizes for each active intervention group in multi-arm trials, allowing for more accurate inclusion of available data. Additionally, we conducted meta-regression analyses to explore potential sources of effect size variability. Results indicated that studies implementing ITT analyses and those using sham as a comparison were significantly associated with differences in treatment effects on motor symptoms in PD. Lastly, our risk of bias assessment indicates that the vast majority of included studies were of good methodological quality.

Interpreting results and making comparisons across studies in this systematic review is inherently challenging due to the considerable complexity and variability of factors influencing treatment effects on motor symptoms in PD. Major sources of heterogeneity include differences in medication state (ON versus OFF periods) during assessment which are known to substantially alter UPDRS3 scores and treatment responsiveness (84, 85). Additionally, variability in follow-up duration and disease chronicity further complicate synthesis; longer-term studies may capture progression or adaptation effects not present in near or short-term trials (86). Similar challenges have been highlighted in other systematic reviews and meta-analyses of neuromodulation and pharmacologic interventions in PD, where inconsistency in timing of assessments and population characteristics contributed to heterogeneity in reported outcomes (20). To address these challenges and enhance comparability, we focused our primary analyses on outcomes assessed during the ON medication state and within a near-term follow-up duration (<4 months), reflecting the most common reporting patterns among included studies.

The present meta-analysis demonstrates a statistically significant, though modest, benefit of active MDBN interventions for improving motor symptoms in PD. The pooled effect size (SMD = −0.18, 95% CI [−0.32, −0.05]) indicates that, while the benefit is small, the effect is nonetheless consistent and robust across a diverse set of trials. When estimated in UPDRS3 units, the effect corresponded to an approximate difference of ~2.16 points. Importantly, this magnitude falls at the low end of the range of many commonly cited minimal clinically important difference (MCID) thresholds for improvements in UPDRS3 measures (87–91), suggesting that the average improvement may not meet conventional criteria for a clinically meaningful change at the individual patient level. Nonetheless, such effects may still be relevant at the population level and may contribute to meaningful benefit in selected patients or subgroups (92), particularly when considered as part of a multimodal management strategy. We caution readers that back-transformed UPDRS3 point estimates are provided for clinical context only and should not be interpreted as exact raw-score treatment effects. Our findings are consistent with those of a recent meta-analytic overview of systematic reviews (17), which reported moderate yet significant improvements in motor symptoms with rTMS compared to sham stimulation. The observed moderate-to-substantial heterogeneity further underscores the influence of variability in study design, patient characteristics, and intervention protocols on treatment response. Importantly, this study adds new evidence to the field by synthesizing outcomes across a large number of effect sizes and rigorously accounting for statistical non-independence, providing a more reliable estimate of MDBN efficacy. While the clinical impact may be limited for individual patients, these results highlight a meaningful therapeutic role for neuromodulation as part of a comprehensive management strategy in PD.

Our meta-regression analysis indicates that methodological factors, particularly analysis strategy (ITT) and the use of a sham comparator, may significantly influence treatment effect estimates. Studies employing ITT analysis demonstrated significantly larger treatment effects, whereas those using a sham comparator group reported smaller effects relative to studies without sham controls. These findings likely reflect methodological variations across studies. Analytical choices, such as ITT versus per-protocol analysis, can systematically affect effect sizes (93). ITT analyses frequently require imputation for missing data, and the choice of imputation method can introduce bias, particularly when data are not missing at random, sometimes favoring positive outcomes (94). Additionally, modified ITT analysis practices and publication bias may contribute; for instance, a BMJ review found that RCTs deviating from standard ITT reporting (e.g., post-randomization exclusions) tended to show larger intervention effects than those adhering strictly to ITT (95). Trials with both larger effects and ITT reporting may also be preferentially published (94). Future trials should rigorously adhere to ITT principles and clearly report methods for handling missing data to improve the reliability and interpretability of neuromodulation efficacy estimates. Regarding the use of sham controls, one potential explanation for our finding is the well-established concept that placebo effects can inflate treatment estimates in studies lacking adequate control conditions (96). Therefore, sham comparators more effectively account for placebo responses and expectancy effects, producing more accurate, but often attenuated, estimates of the true treatment effect.

Our analysis identified a discrepancy between the results of the sensitivity analysis and the meta-regression concerning the use of the MDS-UPDRS. Different methodological and analytical factors may explain this finding. The meta-regression adjusts for multiple study-level moderators, therefore it may have been underpowered to detect a significant moderating effect of the outcome scale, particularly because the number of studies using the MDS-UPDRS was limited (only 6 studies). Beyond limited statistical power, other factors may contribute to this discrepancy. Differences between the UPDRS and MDS-UPDRS, including revised scoring anchors, construct coverage (84, 97), and measurement properties (97), may influence sensitivity to motor change and contribute to variability in effect size estimates across studies. In addition, the MDS-UPDRS scale has been adopted more recently and reflects expanded construct coverage; as a result trials using it may differ from earlier UPDRS-based trials in design features, patient populations, or outcomes assessed (98). Finally, given the relatively small number of studies employing the MDS-UPDRS, the observed difference may also reflect random variation rather than a true scale-related effect (99). In contrast, the sensitivity analysis specifically isolates the impact of the outcome measure, which may accentuate differences not evident when confounding factors are accounted for in meta-regression. Differences in study selection, random variation, and the potential for the MDS-UPDRS to be more sensitive to treatment effects in specific contexts (84, 100) may also contribute to these inconsistent results.

The sensitivity analysis comparing invasive and non-invasive neuromodulation modalities revealed comparable effect sizes across intervention types, with greater consistency observed in studies employing invasive techniques, where both intervention types produced significant, though modest, effects on motor symptoms. Although invasive techniques such as DBS are generally expected to provide greater efficacy due to more direct neural targeting, recent RCTs have shown more modest improvements than those reported in earlier studies, particularly over extended follow-up (20, 101). This likely reflects advances in trial methodology (e.g., explicit reporting of randomization and allocation concealment, blinded outcome assessment, prospective trial registration, and longer follow-up durations) and more balanced reporting of outcomes (e.g., inclusion of additional outcomes rather than only motor improvements) across both invasive and non-invasive modalities. Improvements in non-invasive techniques, such as refinements in non-invasive stimulation protocols and trial designs, and more homogeneous patient selection may also help explain the diminishing gap in efficacy. Additionally, the greater consistency observed among invasive neuromodulation studies (*I*^2^ = 0%) likely reflects the well-established protocols, standardized surgical procedures, and more homogeneous patient populations typical of interventions such as DBS. Previous reviews have noted that DBS studies are often conducted in specialized centers with standardized operative and follow-up procedures, which may reduce sources of variability (20). In contrast, non-invasive modalities such as rTMS and tDCS are characterized by a wide range of stimulation parameters, target sites, and participant characteristics, which may account for the increased heterogeneity in effect sizes reported in the literature (102, 103). Importantly, while non-invasive neuromodulation (as compared to invasive) may be associated with slightly smaller or more variable effect sizes, its favorable safety profile and non-invasive nature may make it particularly advantageous in clinical contexts where surgical risk, disease stage, or patient preference limit the use of invasive therapies.

This study has limitations. As discussed above, the interpretation of results is complicated by the considerable complexity and variability of factors influencing treatment effects on motor symptoms in PD, including differences in medication state, disease duration, and follow-up periods. First, our search strategy was limited to RCTs that reported sufficient statistical detail (e.g., mean values with standard deviations) for UPDRS3 outcomes, resulting in the exclusion of studies with incomplete or inconsistently reported data. This may have introduced selection bias and limited the generalizability of our findings. Second, demographic, physical function, and psychosocial data, such as race, functional independence level, and depression severity, were inconsistently reported across studies, preventing meaningful subgroup or meta regression analyses. These variables may impact response to intervention (104–106). Third, the sensitivity analysis findings for long-term follow-up are based solely on invasive interventions, underscoring the need for future studies to evaluate non-invasive device-based interventions over similar timeframes. Fourth, excluding crossover trials may limit generalizability, as such designs are common in neuromodulation studies and capture within-subject effects not reflected in parallel-group trials. Fifth, back-transformed UPDRS3 point estimates are intended to aid clinical interpretation and should not be interpreted as exact raw-score treatment effects. Sixth, although meta-regression identified analysis approach and comparator type as significant moderators, residual heterogeneity likely reflects intervention characteristics that could not be evaluated in meta-regression due to limited statistical power; this represents a limitation of the present analysis and warrants confirmation in future studies. Additionaly, while other neuromodulation approaches, including for example transcutaneous VNS (107) and electrosonic stimulation (ESStim) techniques (108), have demonstrated efficacy in past studies, they were not included because they fell outside the predefined clinical inclusion criteria.Furthermore, this review was not designed as a comprehensive synthesis of any specific individual neuromodulation intervention, and the search strategy was therefore not optimized to exhaust all specific terminology; as a result, some eligible studies may not have been captured (e.g., transcutaneous auricular vagus nerve stimulation (taVNS), repetitive spinal magnetic stimulation (rSMS)), reflecting inherent sensitivity–precision trade-off in the predefined search strategy design (109). All these considerations underline the need for cautious interpretation of our results.

This meta-analysis provides a comprehensive synthesis of current evidence regarding the efficacy of MDBN interventions for motor symptoms in PD. Our findings underscore the importance of methodological rigor and standardization in both trial design and reporting, as analytical choices and comparator selection can meaningfully impact observed treatment effects. Despite the inherent complexity and heterogeneity across studies, both invasive and non-invasive neuromodulation modalities demonstrated significant but modest benefits, with evolving methodologies narrowing the gap in efficacy between these approaches. The high overall quality of included studies adds confidence to our conclusions, yet limitations related to reporting and data availability highlight the ongoing need for more comprehensive and standardized research in this area. Future trials should prioritize complete reporting, stratified analysis based on key influential factors of motor symptoms, and direct comparisons of neuromodulation strategies to further clarify their respective roles in the management of PD.

## Data Availability

The original contributions presented in the study are included in the article/Supplementary material, further inquiries can be directed to the corresponding author.

## Footnotes

1
ppcr.org
