# Supplementary material for: Medical device-based neuromodulation for motor symptoms in Parkinson’s disease: a systematic review and meta-analysis
Source: Front Neurol. 2026 Feb 12;17:1731885. doi: 10.3389/fneur.2026.1731885 (PMC12937557; doi:10.3389/fneur.2026.1731885)
Supplement: Supplementary file 1 [file Table_1.docx]

**Supplementary table 1**. Summary of the included randomized clinical trials.

| **Author** | **Year** | **Country** | **Type of medical device** | **Intervention groups (n)** | **Age years (Mean ± SD)** | **Duration of symptoms years (Mean ± SD)** | **Hoen & Yahr score (Mean ± SD)** | **Neural Target  Location for Stimulation** | **Intervention Schedule (Total # sessions)** | **Combined intervention** | | **N º of follow-ups for UPDRS-III outcomes** | **UPDRS - III Scores - L-Dopa ON State** | | **UPDRS - III Scores - L-Dopa OFF State** | |
| --- | --- | --- | --- | --- | --- | --- | --- | --- | --- | --- | --- | --- | --- | --- | --- | --- |
|  |  |  |  |  |  |  |  |  |  | **Use of combined intervention** | **Type of combined intervention** |  | **UPDRS-III scores  Baseline (Mean ± SD)** | **UPDRS-III scores  Last available follow-up (Mean ± SD)** | **UPDRS-III scores  Baseline (Mean ± SD)** | **UPDRS-III scores  Last available follow-up (Mean ± SD)** |
|  |  |  |  |  |  |  |  |  |  |  |  |  |  |  |  |  |
|  |  |  |  |  |  |  |  |  |  |  |  |  |  |  |  |  |
| **Aftanas** | 2021 | Russia | rTMS | 10Hz rTMS (23) Sham (23) | 10Hz rTMS (63.7± 8.8) Sham (62.9 ± 7.1) | 10Hz rTMS (7± 4) Sham (5.6 ± 4) | 10Hz rTMS (3 ± 1) Sham (3 ± 1) | M1 and Prefrontal Cortex | 10Hz rTMS (20) Sham (20) | 10Hz rTMS (None) Sham (None) | 10Hz rTMS (N/A) Sham (N/A) | 3 follow-ups | 10Hz rTMS (23.4 ± 9.1) Sham (22.4 ± 8.3) | 10Hz rTMS (13.7 ± 7.4) Sham (20.2 ± 6.1) | 10Hz rTMS (N/A) Sham (N/A) | 10Hz rTMS (N/A) Sham (N/A) |
| **Benninger** | 2011 | USA | rTMS | Intermittent Theta-Burst rTMS (13) Sham (13) | Intermittent Theta-Burst rTMS (62.1 ± 6.9) Sham (65.9 ± 9) | Intermittent Theta-Burst rTMS (10.8 ± 7.1) Sham (6.5 ± 3.4) | Intermittent Theta-Burst rTMS (2.6 ± 0.2) Sham (2.5 ± 0.1) | Bilateral M1 and Dorsolateral Pre-frontal Cortex (DLPFC) | Intermittent Theta-Burst rTMS (8) Sham (8) | Intermittent Theta-Burst rTMS (None) Sham (None) | Intermittent Theta-Burst rTMS (None) Sham (None) | 2 follow-ups | Intermittent Theta-Burst rTMS (32 ± 12.86) Sham (37.54 ± 12.86) | Intermittent Theta-Burst rTMS (29.77 ± 11.47) Sham (35.38 ± 11.47) | Intermittent Theta-Burst rTMS (49 ± 12.88) Sham (45.69 ± 12.38) | Intermittent Theta-Burst rTMS (43.25 ± 10.74) Sham (44.69 ± 10.32) |
| **Benninger** | 2012 | Switzerland | rTMS | 50Hz rTMS (13) Sham (13) | 50Hz rTMS (64.5 ± 9.1) Sham (63.7 ± 8.3) | 50Hz rTMS (8.6 ± 4.1) Sham (9.3 ± 6.8) | 50Hz rTMS (2.4 ± 0.2) Sham (2.5 ± 0.3) | Bilateral M1 | 50Hz rTMS (8) Sham (8) | 50Hz rTMS (None) Sham (None) | 50Hz rTMS (None) Sham (None) | 2 follow-ups | 50Hz rTMS (32.8 ± 2.5) Sham (30 ± 2.6) | 50Hz rTMS (30 ± 2.2) Sham (29.25 ± 2.5) | 50Hz rTMS (38.46 ± 2.2) Sham (37.25 ± 2.3) | 50Hz rTMS (36.39 ± 2.1) Sham (35.42 ± 2.2) |
| **Benninger** | 2010 | USA | tDCS | tDCS (13) Sham (12) | tDCS (63.6 ± 9) Sham (64.2 ± 8.8) | tDCS (10.6 ± 7.1) Sham (9.1 ± 3.3) | tDCS (2.4 ± 0.2) Sham (2.5 ± 0.1) | M1 and Prefrontal Cortex | tDCS (8) Sham (8) | tDCS (None) Sham (None) | tDCS (None) Sham (None) | 3 follow-ups | tDCS (22.2 ± 8.7) Sham (17.5 ± 8) | tDCS (23.1 ± 7.8) Sham (17.6 ± 8.5) | tDCS (34 ± 10) Sham (26.5 ± 8.4) | tDCS (34 ± 10.3) Sham (27.1 ± 10.5) |
| **Blomstedt** | 2018 | Sweden | DBS | DBS-cZi Caudal zona incerta (9) Optimal Drug Therapy (10) | DBS-cZi Caudal zona incerta (57 ± 11.4) Optimal Drug Therapy (60.9 ± 9.2) | DBS-cZi Caudal zona incerta (6.4 ± 3) Optimal Drug Therapy (10.3 ± 5.6) | DBS-cZi Caudal zona incerta (N.R.) Optimal Drug Therapy (N.R.) | Caudal zona incerta | N/A | DBS-cZi Caudal zona incerta (None) Optimal Drug Therapy (None) | DBS-cZi Caudal zona incerta (None) Optimal Drug Therapy (None) | 1 follow-up | DBS-cZi Caudal zona incerta (19.4 ± 12.5) Optimal Drug Therapy (20.6 ± 11.9) | DBS-cZi Caudal zona incerta (18.5 ± 12.4) Optimal Drug Therapy (21.8 ± 13.4) | DBS-cZi Caudal zona incerta (33.2 ± 11.4) Optimal Drug Therapy (42.4 ± 14.5) | DBS-cZi Caudal zona incerta (19.5 ± 7.8) Optimal Drug Therapy (37.2 ± 12.2) |
| **Brys** | 2016 | USA | rTMS | 10Hz rTMS Bilateral M1 (14) 10Hz rTMS DPLFC (12) 10Hz rTMS Bilateral M1 + DPLFC (20) Double-Sham (15) | 10Hz rTMS Bilateral M1 (59.6 ± 12.6) 10Hz rTMS DPLFC (64.6 ± 12.3) 10Hz rTMS Bilateral M1 + DPLFC (64.9 ± 8) (Double-Sham): 64 ± 7.4 | 10Hz rTMS Bilateral M1 (8.4 ± 5.2) 10Hz rTMS DPLFC (7.7 ± 4.2) 10Hz rTMS Bilateral M1 + DPLFC (7.3 ± 5.6)  (Double-Sham): 4.5 ± 2.2 | 10Hz rTMS Bilateral M1 (2 ± 0.35) 10Hz rTMS DPLFC (2.9 ± 0.87) 10Hz rTMS Bilateral M1 + DPLFC (2.46 ± 0.66) (Double-Sham): 2.22 ± 0.44 | Bilateral M1 and DLPFC | 10Hz rTMS Bilateral M1 (10) 10Hz rTMS DPLFC (10) 10Hz rTMS Bilateral M1 + DPLFC (10) (Double-Sham): (10) | 10Hz rTMS Bilateral M1: (None) 10Hz rTMS DPLFC (None) 10Hz rTMS Bilateral M1 + DPLFC (None) (Double-Sham): (None) | 10Hz rTMS Bilateral M1 (N/A) 10Hz rTMS DPLFC (N/A) 10Hz rTMS Bilateral M1 + DPLFC (N/A) (Double-Sham): (N/A) | 4 follow-ups | 10Hz rTMS Bilateral M1 (33.1 ± 7.8) 10Hz rTMS DPLFC (32.8 ± 10.7) 10Hz rTMS Bilateral M1 + DPLFC (32.3 ± 8.9)  (Double-Sham): (28.9 ± 6.4) | 10Hz rTMS Bilateral M1 (29.3 ± 11.4) 10Hz rTMS DPLFC (28.1 ± 9.1) 10Hz rTMS Bilateral M1 + DPLFC (30.1 ± 9.4) (Double-Sham): (28.6 ± 7.3) | 10Hz rTMS Bilateral M1 (N/A) 10Hz rTMS DPLFC (N/A) 10Hz rTMS Bilateral M1 + DPLFC (N/A) (Double-Sham): (N/A) | 10Hz rTMS Bilateral M1 (N/A) 10Hz rTMS DPLFC (N/A) 10Hz rTMS Bilateral M1 + DPLFC (N/A) (Double-Sham): (N/A) |
| **Burchiel** | 1999 | USA | DBS | DBS-GPi (4) DBS-SN (6) | DBS-GPi (46.5 ± 11) DBS-SN (62.8 ± 12) | DBS-GPi (10.6 ± 2) DBS-SN (13.6 ± 5) | DBS-GPi (2.5 ± 1) DBS-SN (2.3 ± 1) | DBS (Globus Palidus interna; Subthalamic Nucleus) | N/A | DBS-GPi (None) DBS-SN (None) | DBS-GPi (N/A) DBS-SN (N/A) | 4 follow-ups | DBS-GPi (38 ± 8.28) DBS-SN (23.2 ± 7.49) | DBS-GPi (22.3 ± 8.51) DBS-SN (22.4 ± 9.55) | DBS-GPi (68 ± 24.7) DBS-SN (49 ± 14.8) | DBS-GPi (46.1 ± 14.67) DBS-SN (33.1 ± 13.32) |
| **Celiker** | 2019 | Turkey | DBS | DBS-GPi (7) DBS-SN (6) | DBS-GPi (54 ± 4.51) DBS-SN (56.16 ± 9.6) | DBS-GPi (10.16 ± 3.12) DBS-SN (9 ± 2) | DBS-GPi (3.16 ± 0.4) DBS-SN (3 ± 0.89) | DBS (Globus Palidus interna; Subthalamic Nucleus) | N/A | DBS-GPi (None) DBS-SN (None) | DBS-GPi (N/A) DBS-SN (N/A) | 3 follow-ups | DBS-GPi (22.5 ± 6.65) DBS-SN (22.16 ± 6.55) | DBS-GPi (12.2 ± --) DBS-SN (11.4 ± --) | DBS-GPi (49 ± 13.57) DBS-SN (47 ± 14.01) | DBS-GPi (N/A) DBS-SN (N/A) |
| **Chang** | 2017 | South Korea | rTMS + tDCS | rTMS + tDCS (16) rTMS + Sham tDCS (16) | rTMS + tDCS (63.6 ± 7.5) rTMS + Sham tDCS (63.8 ± 8.3) | rTMS + tDCS (9.8 ± 4.7) rTMS + Sham tDCS (9.1 ± 5.3) | rTMS + tDCS (2.5 ± 1) rTMS + Sham tDCS (2.5 ± 0.88) | Bilateral M1 | rTMS + tDCS (5) rTMS + Sham tDCS (5) | rTMS + tDCS (Yes) rTMS + Sham tDCS (None) | rTMS + tDCS (Device-Based rTMS + tDCS) rTMS + Sham tDCS (N/A) | 2 follow-ups | rTMS + tDCS (9.9 ± 4.6) rTMS + Sham tDCS (12.9 ± 7.4) | rTMS + tDCS (7.4 ± 4.5) rTMS + Sham tDCS (10.3 ± 7.9) | rTMS + tDCS (N/A) rTMS + Sham tDCS (N/A) | rTMS + tDCS (N/A) rTMS + Sham tDCS (N/A) |
| **Charles** | 2014 | USA | DBS | Bilateral STN + Optimal drug treatment (15) Optimal drug treatment (15) | Bilateral STN + Optimal drug treatment (60 ± 6.8) Optimal drug treatment (60 ± 7) | N/A | Bilateral STN + Optimal drug treatment (1.7 ± 0.5) Optimal drug treatment (1.8 ± 0.4) | DBS-SN (Subthalamic Nucleus) | N/A | Bilateral STN + Optimal drug treatment (None) Optimal drug treatment (None) | Bilateral STN + Optimal drug treatment (N/A) Optimal drug treatment (N/A) | 4 follow-ups | Bilateral STN + Optimal drug treatment (23.7 ± 12.3) Optimal drug treatment (21.3 ± 9.2) | Bilateral STN + Optimal drug treatment (23.7 ± 13.7) Optimal drug treatment (24.4 ± 9.5) | Bilateral STN + Optimal drug treatment (28 ± 10.2) Optimal drug treatment (29.5 ± 8.7) | Bilateral STN + Optimal drug treatment (36.1 ± 13.3) Optimal drug treatment (39.1 ± 9.1) |
| **Chung** | 2020 | China | rTMS | 1Hz rTMS (17) 25Hz-rTMS (17) Sham (17) | 1Hz rTMS (62.1 ± 5.7) 25Hz rTMS (62.7 ± 6.8) Sham (62.1 ± 5.7) | 1Hz rTMS (7.5 ± 4.9) 25Hz rTMS (5.2 ± 3.4) Sham (6.9 ± 3.3) | 1Hz rTMS (2.2 ± 0.4) 25Hz rTMS (2.2 ± 0.3) Sham (2.3 ± 0.3) | M1 (Bilateral) | 1Hz rTMS (12) 25Hz rTMS (12) Sham (12) | 1Hz rTMS (Yes) 25Hz rTMS (Yes) Sham (Yes) | 1Hz rTMS (Exercise-based; 30-minute treadmill training) 25Hz rTMS (Exercise-based; 30-minute treadmill training) Sham (Exercise-based; 30-minute treadmill training) | 3 follow-ups | 1Hz rTMS (27.1 ± 9.6) 25Hz rTMS (27.9 ± 10.5) Sham (27.9 ± 10.6) | 1Hz rTMS (21.9 ± 7.8) 25Hz rTMS (22.5 ± 10.1) Sham (27.3 ± 8.0) | 1Hz rTMS (N/A) 25Hz rTMS (N/A) Sham (N/A) | 1Hz rTMS (N/A) 25Hz rTMS (N/A) Sham (N/A) |
| **Cohen** | 2018 | Israel | rDTMS | rDTMS (26) Sham (22) | rDTMS (64.4 ± 6.8) Sham (66.8 ± 8.1) | rDTMS (4.7 ± 3.4) Sham (5.6 ± 3.7) | rDTMS (2 ± 0.5) Sham (2 ± 0.5) | M1 and Prefrontal Cortex | rDTMS (1) Sham (1) | rDTMS (None) Sham (None) | rDTMS (N/A) Sham (N/A) | 1 follow-up | rDTMS (29.4 ± 7.12) Sham (28.6 ± 6.56) | rDTMS (27.1 ± 5.6) Sham (27.6 ± 5.97) | rDTMS (N/A) Sham (N/A) | rDTMS (N/A) Sham (N/A) |
| **Costa-Ribeiro** | 2017 | Brazil | tDCS | tDCS +Cueing Gait Training (12) Sham (12) | tDCS +Cueing Gait Training (61.1 ± 9.1) Sham (62 ± 16.7) | tDCS +Cueing Gait Training (6.1 ± 3.8) Sham (6.3 ± 3.7) | tDCS +Cueing Gait Training (2.36 ± 0.71) Sham (2.32 ± 0.68) | Supplementary Motor Area | tDCS +Cueing Gait Training (10) Sham (10) | tDCS +Cueing Gait Training (Yes) Sham (Yes) | tDCS +Cueing Gait Training (Motor-Learning Based through visual cueing training) Sham (Motor-Learning Based through visual cueing training) | 2 follow-ups | tDCS +Cueing Gait Training (19 ± 4.9) Sham (17.6 ± 5.1) | tDCS +Cueing Gait Training (11 ± 5) Sham (11 ± 5.8) | tDCS +Cueing Gait Training (N/A) Sham (N/A) | tDCS +Cueing Gait Training (N/A) Sham (N/A) |
| **delOlmo** | 2007 | Spain | rTMS | 10Hz rTMS (8) Sham (5) | N/A | N/A | N/A | Dorso-Lateral Prefrontal Cortex (DLPFC) | 10Hz rTMS (10) Sham (10) | 10Hz rTMS (None) Sham (None) | 10Hz rTMS (N/A) Sham (N/A) | 1 follow-up | 10Hz rTMS (27.05 ± 4.24) Sham (26.72 ± 4.19) | 10Hz rTMS (25.98 ± 5.35) Sham (26.44 ± 5.02) | 10Hz rTMS (N/A) Sham (N/A) | 10Hz rTMS (N/A) Sham (N/A) |
| **Deuschl** | 2006 | Germany | DBS | DBS-SN (78) Drug Therapy (78) | DBS-SN (60.5 ± 7.4) Drug Therapy (60.8 ± 7.8) | N/A | DBS-SN (4 ± 1) Drug Therapy (4 ± 1) | DBS-SN (Subthalamic Nucleus) | N/A | DBS-SN (None) Drug Therapy (None) | DBS-SN (N/A) Drug Therapy (N/A) | 1 follow-up | DBS-SN (18.9 ± 9.3) Drug Therapy (17.3 ± 9.6) | DBS-SN (14.6 ± 8.5) Drug Therapy (17.5 ± 10.6) | DBS-SN (48 ± 12.3) (Drug Therapy): (46.8 ± 12.1) | DBS-SN (28.3 ± 14.7) Drug Therapy (46 ± 12.6) |
| **Esselink** | 2006 | Netherlands | DBS | DBS-SN (20) Unilateral pallidotomy (14) | DBS-SN (61 ± 11) Unilateral pallidotomy (62 ± 11) | DBS-SN (12 ± 8) Unilateral pallidotomy (11 ± 7) | N/A | DBS-SN (Subthalamic Nucleus) | N/A | DBS-SN (None) Unilateral pallidotomy (None) | DBS-SN (N/A) (Unilateral pallidotomy): N/A | 2 follow-ups | DBS-SN (21 ± 14) Unilateral pallidotomy (15.5 ± 13) | DBS-SN (11.5 ± 47) Unilateral pallidotomy (22 ± 34) | DBS-SN (51.5 ± 16) Unilateral pallidotomy (46.5 ± 27) | DBS-SN (24 ± 50) Unilateral pallidotomy (32 ± 40) |
| **Grobe-Einsler** | 2024 | Germany | rTMS | 48Hz rTMS (20) Sham (16) | 48Hz rTMS (68 ± 11.3) Sham (71 ± 11.3) | N/A | 48Hz rTMS (2 ± 2) Sham (1.5 ± 2) | Cerebellum | 48Hz rTMS (15) Sham (15) | 48Hz rTMS (Yes) Sham (None) | 48Hz rTMS (Motor-Based Learning with a Multimodal Physiotherapy Protocol) Sham (N/A) | 2 follow-ups | 48Hz rTMS (30.65 ± 23.7) Sham (28.94 ± 16.5) | 48Hz rTMS (17.10 ± 7.58) Sham (30.7 ± 17.2) | N/A | N/A |
| **Grobe-Einsler** | 2024 | Germany | rTMS | 50Hz rTMS (18) Sham (17) | 50Hz rTMS (66.06 ± 9.70) Sham (70.41 ± 13.37) | N/A | 50Hz rTMS (2.11 ± 0.90) Sham (1.94 ± 0.75) | Cerebellum | 50Hz rTMS (10) Sham (10) | 50Hz rTMS (None) Sham (None) | 50Hz rTMS (N/A) Sham (N/A) | 2 follow-ups | 50Hz rTMS (34.17 ± 14.02) Sham (30.12 ± 14.65) | 50Hz rTMS (30.53 ± 12.87) Sham (32.92 ± 12.59) | N/A | N/A |
| **Hacker** | 2018 | USA | DBS | DBS + Optimal drug treatment (14) Optimal drug treatment (14) | DBS + Optimal drug treatment (61.3 ± 6.4) Optimal drug treatment (60.5 ± 6.6) | DBS + Optimal drug treatment (1.9 ± 1.5) Optimal drug treatment (2.1 ± 1.6) | DBS + Optimal drug treatment (2.0 ± 1.4) Optimal drug treatment (1.9 ± 1.0) | DBS-SN (Subthalamic Nucleus) | N/A | DBS + Optimal drug treatment (None) Optimal drug treatment (None) | DBS + Optimal drug treatment (N/A) Optimal drug treatment (N/A) | 4 follow-up | DBS + Optimal drug treatment (24.8 ± 12) Optimal drug treatment (21.3 ± 9.2) | DBS + Optimal drug treatment (25.1 ± 13.2) Optimal drug treatment (24.4 ± 9.5) | DBS + Optimal drug treatment (29.2 ± 9.5) Optimal drug treatment (29.5 ± 8.7) | DBS + Optimal drug treatment (37.7 ± 28.44) Optimal drug treatment (39.1 ± 14.97) |
| **Hacker** | 2020 | USA | DBS | DBS + Optimal drug treatment (14) Optimal drug treatment (14) | DBS + Optimal drug treatment (N/A) Sham (N/A) | DBS + Optimal drug treatment (N/A) Sham (N/A) | DBS + Optimal drug treatment (N/A) Sham (N/A) | DBS-SN (Subthalamic Nucleus) | N/A | DBS + Optimal drug treatment (None) Sham (None) | DBS + Optimal drug treatment (N/A) Sham (N/A) | 5 follow-ups | DBS + Optimal drug treatment (24.8 ± 12) Sham (21.3 ± 9.2) | DBS + Optimal drug treatment (26.5 ± 11.9) Sham (28.4 ± 9.5) | DBS + Optimal drug treatment (29.2 ± 9.5) Optimal drug treatment (29.5 ± 8.7) | N.R. |
| **Ji** | 2021 | China | rTMS | rTMS with cTBS at SMA (25) Sham (21) | rTMS with cTBS at SMA (61.7 ± 1.57) Sham (60.2 ± 1.97) | rTMS with cTBS at SMA (4.3 ± 0.52) Sham (5.3 ± 0.83) | N/A | TMS with Continuous theta-burst stimulation (cTBS) at supplementary motor area (SMA) | rTMS with cTBS at SMA (14) Sham (14) | rTMS with cTBS at SMA (None) Sham (None) | rTMS with cTBS at SMA (N/A) Sham (N/A) | 2 follow-ups | rTMS with cTBS at SMA (28 ± 9.94) Sham (29.3 ± 9.08) | rTMS with cTBS at SMA (20.6 ± 8.54) Sham (30.4 ± 9.66) | N/A | N/A |
| **Khedr** | 2019 | Egypt | rTMS | 20Hz rTMS (26) 1Hz rTMS (26) | 20Hz rTMS (59.58 ± 11.28) 1Hz rTMS (55.88 ± 13.84) | 20Hz rTMS (4.6 ± 3.64) 1Hz rTMS (3.39 ± 4.73) | N/A | Bilateral M1 | 20Hz rTMS (10) 1Hz rTMS (10) | 20Hz rTMS (None) 1Hz rTMS (None) | 20Hz rTMS (N/A) 1Hz rTMS (N/A) | 2 follow-ups | N/A | N/A | 20Hz rTMS (45.27 ± 20.01) 1Hz rTMS (46.46 ± 22.37) | 20Hz rTMS (34.54 ± 13.02) 1Hz rTMS (42.62 ± 25.69) |
| **Lee** | 2021 | South Korea | tDCS | Anodal tDCS (16) Sham (16) | Anodal tDCS (70 ± 3.76) Sham (71.33 ± 3.27) | Anodal tDCS (0.52 ± 0.08) Sham (0.58 ± 0.11) | Anodal tDCS (2.47 ± 0.52) Sham (2.8 ± 0.41) | Bilateral M1 | Anodal tDCS (20) Sham (20) | Anodal tDCS (Yes) Sham (Yes) | Anodal tDCS (Motor-Learning based) Sham (Motor-Learning based) | 2 follow-ups | Anodal tDCS (34.2 ± 7.82) Sham (38.67 ± 9.6) | Anodal tDCS (25.2 ± 8.99) Sham (32.6 ± 8.7) | N/A | N/A |
| **Li** | 2017 | China | DBS | Stimulation begins after 1 month after surgery (32) Stimulation begins after 3 months after surgery (32) | Stimulation begins after 1 month after surgery (55.47 ± 9.13) Stimulation begins after 3 months after surgery (56.88 ± 9.89) | Stimulation begins after 1 month after surgery (9.8 ± 4.47) Stimulation begins after 3 months after surgery (8.82 ± 2.9) | Stimulation begins after 1 month after surgery (3.22 ± 0.53) Stimulation begins after 3 month after surgery (3.31 ± 0.52) | DBS-SN (Subthalamic Nucleus) | N/A | Stimulation begins after 1 month after surgery (None) Stimulation begins after 3 month after surgery (None) | Stimulation begins after 1 month after surgery (N/A) Stimulation begins after 3 month after surgery (N/A) | 3 follow-ups | Stimulation begins after 1 month after surgery (21.66 ± 11.38) Stimulation begins after 3 month after surgery (20.56 ± 9.87) | Stimulation begins after 1 month after surgery (13.74 ± 8.04) Stimulation begins after 3 month after surgery (12.73 ± 9.28) | Stimulation begins after 1 month after surgery (50.98 ± 12.84) Stimulation begins after 3 month after surgery (49.64 ± 11.37) | Stimulation begins after 1 month after surgery (18.69 ± 9.66) Stimulation begins after 3 month after surgery (17.47 ± 9.23) |
| **Li** | 2020 | China | rTMS | 20Hz rTMS (24) Sham (24) | 20Hz rTMS (61.67 ± 6.92) Sham (61.46 ± 8.4) | 20Hz rTMS (5.48 ± 3.69) Sham (6.46 ± 5.17) | 20Hz rTMS (1.85 ± 0.63) Sham (1.83 ± 0.64) | Bilateral M1 | 20Hz rTMS (5) Sham (5) | 20Hz rTMS (None) Sham (None) | 20Hz rTMS (N/A) Sham (N/A) | 3 follow-ups | 20Hz rTMS (27.79 ± 16.35) Sham (28.38 ± 15.35) | 20Hz rTMS (25.92 ± 16.35) Sham (28.71 ± 16.35) | N/A | N/A |
| **Liao** | 2024 | China | rTMS | 0.5Hz rTMS + Ballance Apparatus Training (20) 0.5 Hz TMS (20) Control (20) | 0.5Hz TMS + Ballance Apparatus Training (64.89 ± 9.31) 0.5 Hz TMS (59.95 ± 10.66) Control (62.58 ± 10.34) | 0.5Hz TMS + Ballance Apparatus Training (7.21 ± 3.51) 0.5 Hz TMS (6.45 ± 2.61) Control (6.95 ± 2.54) | 0.5Hz TMS + Ballance Apparatus Training (2.74 ± 0.87) 0.5 Hz TMS (2.75 ± 1.02) Control (2.55 ± 1.05) | Supplementary Motor Area | 0.5Hz TMS + Ballance Apparatus Training (48) 0.5 Hz TMS (48) Control (48) | 0.5Hz TMS + Ballance Apparatus Training (Yes) 0.5 Hz TMS (None) Control (None) | 0.5Hz TMS + Ballance Apparatus Training (Motor-Learning Based) 0.5 Hz TMS (N/A) Control (N/A) | 1 follow-up | 0.5Hz TMS + Ballance Apparatus Training (26.42 ± 2.55) 0.5 Hz TMS (26.05 ± 2.5) Control (25.45 ± 3.27) | 0.5Hz TMS + Ballance Apparatus Training (22.58 ± 2.54) 0.5 Hz TMS (23.55 ± 3.24) Control (20.15 ± 2.87) | N/A | N/A |
| **Makkos** | 2016 | Hungary | rTMS | 5Hz rTMS (23)  Sham (23) | 5Hz rTMS (67 ± 13) Sham (66 ± 8) | 5Hz rTMS (6 ± 7) Sham (5 ± 6) | N/A | Bilateral M1 | 5Hz rTMS (10) Sham (10) | 5Hz rTMS (None) Sham (None) | 5Hz rTMS (N/A) Sham (N/A) | 2 follow-ups | 5Hz rTMS (26 ± 30) Sham (29 ± 26) | 5Hz rTMS (20 ± 17) Sham (27 ± 20) | N/A | N/A |
| **Manenti** | 2018 | Italy | tDCS | tDCS (11) Sham (11) | tDCS (65.5 ± 6.4) Sham (63.8 ± 7.1) | tDCS (6.2 ± 3.9) Sham (7.6 ± 3.4) | tDCS (1.6 ± 0.8) Sham (1.9 ± 0.5) | Left Dorso-Lateral Prefrontal Cortex (DLPFC) | tDCS (10) Sham (10) | tDCS (Yes) Sham (Yes) | tDCS (Cognitive-Training) Sham (Cognitive-Training) | 2 follow-ups | tDCS (26 ± 10.3) Sham (22.7 ± 7.8) | tDCS (24.5 ± 9.7) Sham (22.4 ± 6.3) | N/A | N/A |
| **Martinez-Fernandez** | 2020 | Spain | Focused Subthalamotomy Ultrasound | Focused Subthalamotomy Ultrasound (27) Sham (13) | Focused Subthalamotomy Ultrasound (56.6 ± 9.3) Sham (58.1 ± 8.8) | Focused Subthalamotomy Ultrasound (5.6 ± 2.5) Sham (7.3 ± 3.8) | N/A | DBS-SN (Subthalamic Nucleus) | N/A | Focused Subthalamotomy Ultrasound (None) Sham (None) | Focused Subthalamotomy Ultrasound (N/A) Sham (N/A) | 2 follow-ups | Focused Subthalamotomy Ultrasound (26.9 ± 6.7) Sham (25.1 ± 8.1) | Focused Subthalamotomy Ultrasound (18.2 ± 5.5) Sham (26.2 ± 8.2) | Focused Subthalamotomy Ultrasound (39.9 ± 9.7) Sham (40.1 ± 8.1) | Focused Subthalamotomy Ultrasound (24.7 ± 8) Sham (37.8 ± 9) |
| **Merello** | 2008 | Argentina | DBS and Radiofrequency Lesion Generator | Bilateral STN (6) Bilateral Subthalatomy (5) Unilateral Subthalatomy (5) | Bilateral STN (62.1 ± 3.4) Bilateral Subthalatomy (57.8 ± 3.7) Unilateral Subthalatomy (63 ± 3.8) | Bilateral STN (12.6 ± 1.5) Bilateral Subthalatomy (12.4 ± 1.6) Unilateral Subthalatomy (13.6 ± 1.8) | N/A | DBS (Subthalamic Nucleus) | N/A | Bilateral STN (None) Bilateral Subthalatomy (None) Unilateral Subthalatomy (Yes) | Bilateral STN (N/A) Bilateral Subthalatomy (N/A) Unilateral Subthalatomy (Surgical-Based) | 2 follow-ups | N/A | N/A | Bilateral STN (46 ± 20.19) Bilateral Subthalatomy (50.6 ± 9.76) Unilateral Subthalatomy (54 ± 10.56) | Bilateral STN (18 ± 11.58) Bilateral Subthalatomy (24.2 ± 4.09) Unilateral Subthalatomy (20.6 ± 4.67) |
| **Mi** | 2019 | China | rTMS | 10Hz rTMS (20) Sham (10) | 10Hz rTMS (62.65 ± 10.56) Sham (65.6 ± 8.68) | 10Hz rTMS (9.15 ± 5.82) Sham (7.4 ± 4.83) | 10Hz rTMS (2.6 ± 0.85) Sham (2.35 ± 0.91) | Supplementary Motor Area | 10Hz rTMS (10) Sham (10) | 10Hz rTMS (None) Sham (None) | 10Hz rTMS (N/A) Sham (N/A) | 4 follow-ups | 10Hz rTMS (34.75 ± 13.6) Sham (35.3 ± 16.71) | 10Hz rTMS (28.96 ± 13.91) Sham (35.16 ± 19.64) | N/A | N/A |
| **Mitsui** | 2022 | Japan | rTSMS | 5Hz rTSMS (50) Sham (50) | 5Hz rTSMS (68.66 ± 8.69) Sham (70.98 ± 7.3) | 5Hz rTSMS (7.68 ± 5.44) Sham (5.74 ± 5.05) | 5Hz rTSMS (3.3 ± 0.5) Sham (3.5 ± 0.2) | Spine | 5Hz rTSMS (8) Sham (8) | 5Hz rTSMS (None) Sham (None) | 5Hz rTSMS (N/A) Sham (N/A) | 2 follow-ups | 5Hz rTSMS (46.24 ± 14.58) Sham (45.24 ± 15.01) | 5Hz rTSMS (32.6 ± 13.77) Sham (34.9 ± 10.22) | N/A | N/A |
| **Odekerken** | 2016 | Netherlands | DBS | DBS-GPi (65) DBS-SN (63) | DBS-GPi (59.10 ± 7.8) DBS-SN (60.9 ± 7.6) | DBS-GPi (10.8 ± 4.2) DBS-SN (12 ± 5.3) | DBS-GPi (2.5 ± --) DBS-SN (2.5 ± --) | DBS-GPi (Globus Palidus interna) DBS-SN (Subthalamic Nucleus) | N/A | DBS-GPi (None) DBS-SN (None) | DBS-GPi (N/A) DBS-SN (N/A) | 2 follow-ups | N/A | N/A | DBS-GPi (43 ± 20.5) DBS-SN (41 ± 21.2) | DBS-GPi (33 ± 18) DBS-SN (28 ± 16) |
| **Odekerken** | 2013 | Netherlands | DBS | DBS-GPi (65) DBS-SN (63) | DBS-GPi (59.1 ± 7.8) DBS-SN (60.9 ± 7.6) | DBS-GPi (10.8 ± 4.2) DBS-SN (12 ± 5.3) | DBS-GPi (2.5 ± --) DBS-SN (2.5 ± --) | DBS-GPi (Globus Palidus interna) DBS-SN (Subthalamic Nucleus) | N/A | DBS-GPi (None) DBS-SN (None) | DBS-GPi (N/A) DBS-SN (N/A) | 1 follow-up | DBS-GPi (16 ± 8) DBS-SN (17 ± 9.9) | DBS-GPi (16 ± 9.4) DBS-SN (14.4 ± 11.1) | DBS-GPi (43.8 ± 13.5) DBS-SN (44.4 ± 15.5) | DBS-GPi (32.4 ± 12.6) DBS-SN (24.1 ± 14.4 ) |
| **Okun** | 2014 | USA | DBS | DBS-SN (16) DBS-GPi (14) | DBS-SN (58 ± 10.7) DBS-GPi (60.1 ± 5.5) | DBS-SN (12.1 ± 4.5) DBS-GPi (11.5 ± 3.3) | DBS-SN (2 ± 0.5) DBS-GPi (2 ± 0.5) | DBS-SN (Subthalamic Nucleus) DBS-GPi (Globus Palidus interna) | N/A | DBS-SN (None) DBS-GPi (None) | DBS-SN (N/A) DBS-GPi (N/A) | 4 follow-ups | DBS-SN (21.3 ± 7.56) DBS-GPi (20.8 ± 8.68) | DBS-SN (22.6 ± 8.21) DBS-GPi (22.7 ± 6.56) | DBS-SN (41.2 ± 9.32) DBS-GPi (40.5 ± 11.2) | DBS-SN (29.7 ± 10.4) DBS-GPi (33.9 ± 12.1) |
| **Pal** | 2010 | Hungary | rTMS | 0.5Hz rTMS (12) Sham (10) | 0.5Hz rTMS (68.5 ± 10.5) Sham (67.5 ± 15) | 0.5Hz rTMS (6 ± 6.5) Sham (6.5 ± 6.75) | N/A | Left Dorso-Lateral Prefrontal Cortex (DLPFC) | 0.5Hz rTMS (10) Sham (10) | 0.5Hz rTMS (None) Sham (None) | 0.5Hz rTMS (N/A) Sham (N/A) | 2 follow-ups | 0.5Hz rTMS (23.5 ± 12) Sham (21.5 ± 15) | 0.5Hz rTMS (16 ± 7.5) Sham (19.5 ± 11) | N/A | N/A |
| **Romero** | 2024 | Spain | rTMS | 10Hz rTMS (10) EEG-NFB (11) rTMS + EEG-NFB (10) (Control) : (9) | 10Hz rTMS (64.4 ± 6.38) EEG-NFB (62.18 ± 8.18) rTMS + EEG-NFB (59 ± 7.62) Control (66.89 ± 9.07) | 10Hz rTMS (6 ± 3.06) EEG-NFB (5.91 ± 3.75) rTMS + EEG-NFB (5.2 ± 3.16) Control (6.22 ± 4.12) | 10Hz rTMS (2 ± 0.13) EEG-NFB (2 ± 1.5) rTMS + EEG-NFB (1.5 ± 1) Control (2 ± 0.5) | Bilateral M1 | 10Hz rTMS (8) EEG-NFB (8) rTMS + EEG-NFB (8) Control (0) | 10Hz rTMS (None) EEG-NFB (None) rTMS + EEG-NFB (Yes) Control (None) | 10Hz rTMS (None) EEG-NFB (N/A) rTMS + EEG-NFB (Device-based) Control (N/A) | 2 follow-ups | 10Hz rTMS (17 ± 8.18) EEG-NFB (15.55 ± 7.28) rTMS + EEG-NFB (15.0 ± 6.39) Control (15.67 ± 10.17) | 10Hz rTMS (12.9 ± 27.51) EEG-NFB (13.6 ± 30.75) rTMS + EEG-NFB (10.6 ± 24.41) Control (14.5 ± 22.2) | N/A | N/A |
| **Schuobach** | 2007 | France | DBS | DBS-SN (10) Optimal Drug Therapy (10) | DBS-SN (48.4 ± 3.3) Optimal Drug Therapy (48.5 ± 3) | N/A | N/A | DBS-SN (Subthalamic Nucleus) | N/A | DBS-SN (None) Optimal Drug Therapy (None) | DBS-SN (N/A) Optimal Drug Therapy (N/A) | 3 follow-ups | DBS-SN (3.2 ± 7.11) Optimal Drug Therapy (3.09 ± 4.26) | DBS-SN (2.64 ± 2.49) Optimal Drug Therapy (4.66 ± 9.24) | DBS-SN (32.64 ± 27) Optimal Drug Therapy (25.22 ± 17.05) | DBS-SN (8.93 ± 11.01) Optimal Drug Therapy (30.78 ± 25.22) |
| **Shirota** | 2013 | Japan | rTMS | 1Hz rTMS (36) 10Hz rTMS (36) Sham (36) | 1Hz rTMS (68.8 ± 7.6) 10Hz rTMS (67.9 ± 8.4) Sham (65.7 ± 8.5) | 1Hz rTMS (8.5 ± 7.3) 10Hz rTMS (7.8 ± 6.6) Sham (7.6 ± 4.4) | 1Hz rTMS (3 ± 1) 10Hz rTMS (3 ± 1) Sham (3 ± 1) | Supplementary Motor Area | 1Hz rTMS (8) 10Hz rTMS (8) Sham (8) | 1Hz rTMS (None) 10Hz rTMS (None) Sham (None) | 1Hz rTMS (N/A) 10Hz rTMS (N/A) Sham (N/A) | 4 follow-ups | 1Hz rTMS (24.75 ± 19.36) 10Hz rTMS (23.12 ± 18.89) Sham (24.09 ± 27.06) | 1Hz rTMS (18.77 ± 21.81) 10Hz rTMS (23.68 ± 20.52) Sham (23.3 ± 21.22) | N/A | N/A |
| **Song** | 2024 | China | rTMS | 10Hz rTMS (22) Sham (22) | 10Hz rTMS (67.36 ± 6.99) Sham (70.5 ± 6.76) | 10Hz rTMS (6.18 ± 1.62) Sham (6.77 ± 2.02) | 10Hz rTMS (2.5 ± 1) Sham (2.5 ± 1) | Bilateral M1 | 10Hz rTMS (10) Sham (10) | 10Hz rTMS (None) Sham (None) | 10Hz rTMS (N/A) Sham (N/A) | 2 follow-ups | 10Hz rTMS (41.68 ± 12.96) Sham (43.23 ± 10.73) | 10Hz rTMS (33.23 ± 13.17) Sham (43.54 ± 10.21) | N/A | N/A |
| **Spagnolo** | 2021 | Italy | rTMS | 10Hz rTMS M1+PFC (19) 11Hz rTMS M1 (20) Sham (20) | 10Hz rTMS M1+PFC (63.9 ± 10) 11Hz rTMS M1 (60.4 ± 8.1) Sham (64.2 ± 5.5) | 10Hz rTMS M1+PFC (7.6 ± 4.9) 11Hz rTMS M1 (5.8 ± 2.1) Sham (7.2 ± 3) | 10Hz rTMS M1+PFC (2 ± 0.5) 11Hz rTMS M1 (2 ± 0.01) Sham (2 ± 0.01) | Bilateral M1 Prefrontal Cortex | 10Hz rTMS M1+PFC (12) 11Hz rTMS M1 (12) Sham (12) | 10Hz rTMS M1+PFC (None) 11Hz rTMS M1 (None) Sham (None) | 10Hz rTMS M1+PFC (N/A) 11Hz rTMS M1 (N/A) Sham (N/A) | 1 follow-up | N/A | N/A | 10Hz rTMS M1+PFC (42.4 ± 11.2) 11Hz rTMS M1 (39.1 ± 10) Sham (43.3 ± 9) | 10Hz rTMS M1+PFC (30.5 ± 10.3) 11Hz rTMS M1 (29.5 ± 11.2) Sham (36.8 ± 9.6) |
| **Sun** | 2024 | China | rTMS | 10Hz rTMS (40) 10Hz rTMS + 25Hz rPMS (40) | 10Hz rTMS (63.8 ± 9.87) 10Hz rTMS + 25Hz rPMS (62.62 ± 8.23) | 10Hz rTMS (4.65 ± 2.68) 10Hz rTMS + 25Hz rPMS (4.21 ± 2.19) | N/A | Bilateral M1 | 10Hz rTMS (10) 10Hz rTMS + 25Hz rPMS (10) | 10Hz rTMS (None) 10Hz rTMS + 25Hz rPMS (Yes) | 10Hz rTMS (N/A) 10Hz rTMS + 25Hz rPMS (Device-Based) | 1 follow-up | 10Hz rTMS (25 ± 14) 10Hz rTMS + 25Hz rPMS (22 ± 9) | 10Hz rTMS (18 ± 9) 10Hz rTMS + 25Hz rPMS (14 ± 7) | N/A | N/A |
| **Vitek** | 2020 | USA | DBS | Therapeutic (121) Sub-therapeutic (39) | Therapeutic (60.7 ± 7.9) Sub-therapeutic (57.5 ± 7.7) | Therapeutic (10 ± 3.6) Sub-therapeutic (10.2 ± 3.6) | NR | DBS-SN (Subthalamic Nucleus) | N/A | Therapeutic (None) Sub-therapeutic (None) | Therapeutic (N/A) Sub-therapeutic (N/A) | 1 follow-up | Therapeutic (20.28 ± 9.28) Sub-therapeutic (20.44 ± 12.14) | Therapeutic (15.33 ± 8.26) Sub-therapeutic (17.67 ± 10.68) | Therapeutic (36.88 ± 10.83) Sub-therapeutic (37.74 ±10.4) | Therapeutic (25.12 ± 10.99) Sub-therapeutic (35.49 ± 8.38) |
| **Weaver** | 2012 | USA | DBS | DBS-Gpi (89) DBS-SN (70) | DBS-Gpi (60.4±8.3) DBS-SN (60.7±8.9) | N/A | DBS-Gpi (3.3±0.8) DBS-SN (3.3±0.8) | DBS (Globus Palidus interna) DBS (Subthalamic Nucleus) | N/A | DBS-GPi (None) DBS-SN (None) | DBS-GPi (N/A) DBS-SN (N/A) | 3 follow-ups | DBS-GPi (21±11.4) DBS-SN (21.6±9.1 ) | DBS-GPi (20.4±9.7) DBS-SN (24±11.3 ) | DBS-GPi (41.1±12.2) DBS-SN (42.5±12.4 ) | DBS-GPi (27.1±12.3) DBS-SN (29.7±12.7 ) |
| **Weaver** | 2009 | USA | DBS | DBS (121) Optimal Drug Therapy (134) | DBS (62.4 ± 8.8) Optimal Drug Therapy (62.3 ± 9) | N/A | DBS (3.4 ± 0.9) Optimal Drug Therapy (3.3 ± 0.8) | DBS (either Globus Palidus interna or Subthalamic Nucleus) | N/A | DBS (None) Optimal Drug Therapy (None) | DBS (N/A) Optimal Drug Therapy (N/A) | 2 follow-ups | DBS (22.6 ± 12.6) Optimal Drug Therapy (23.4 ± 11.1) | DBS (20.3 ± 11.3) Optimal Drug Therapy (23.1 ± 11.7) | DBS (43 ± 13.5) Optimal Drug Therapy (43.2 ± 11.3) | DBS (30.7 ± 14.5) Optimal Drug Therapy (41.6 ± 12.7) |
| **Wu** | 2024 | China | rTMS | 1Hz rTMS (37) Sham (37) | 1Hz rTMS (65 ± 15.3) Sham (65 ± 9.5) | 1Hz rTMS (5 ± 5) Sham (5 ± 6.2) | 1Hz rTMS (2 ± 1) Sham (2 ± 1) | Dorso-Lateral Prefrontal Cortex (DLPFC) | 1Hz rTMS (10) Sham (10) | 1Hz rTMS (None) Sham (None) | 1Hz rTMS (N/A) Sham (N/A) | 2 follow-ups | 1Hz rTMS (24 ± 14.5) Sham (22 ± 18.5) | 1Hz rTMS (21.5 ± 14.3) Sham (22 ± 17.5) | N/A | N/A |
| **Zhuang** | 202 | China | rTMS | 10Hz rTMS (19) Sham (14) | 10Hz rTMS (60.58 ± 9.21) Sham (61.57 ± 13.25) | N/A | 10Hz rTMS (2 ± 1) Sham (2.25 ± 1.25) | Dorso-Lateral Prefrontal Cortex (DLPFC) | 10Hz rTMS (10) Sham (10) | 10Hz rTMS (None) Sham (None) | 10Hz rTMS (N/A) Sham (N/A) | 4 follow-ups | 10Hz rTMS (27.84 ± 8.96) Sham (29 ± 13.16) | 10Hz rTMS (27 ± 9.46) Sham (28.79 ± 13.11) | N/A | N/A |
|  |  |  |  |  |  |  |  |  |  |  |  |  |  |  |  |  |
|  |  |  |  |  |  |  |  |  |  |  |  |  |  |  |  |  |
| ***SD****: Standard* *Deviation;* ***rTMS****: Repetitive Transcranial Magnetic Stimulation;* ***tDCS****: Transcranial Direct Current Stimulation;* ***DBS****: Deep Brain Stimulation;* ***cTBS****: Continuous Theta-Burst Stimulation;* ***rTDMS****: Repetitive Deep Transcranial Magnetic Stimulation;* ***rTSMS****: Repetitive Trans-Spinal Magnetic Stimulation;* ***EEG: E****lectroencephalogram;* ***EEG-NFB****: Electroencephalogram–Neurofeedback;* ***Hz****: Hertz;* ***M1****: Primary Motor Cortex;* ***PFC****: Prefrontal Cortex;* ***STN****: Subthalamic Nucleus;* ***cZi****: Caudal Zona Incerta;* ***GPi****: Globus Pallidus interna;* ***DPFC****: Dorso Lateral Pre-Frontal Cortex;* ***DBS-cZi****: Deep Brain Stimulation–Caudal Zona Incerta;* ***DBS-SN****: Deep Brain Stimulation–Subthalamic Nucleus;* ***DBS-GPi****: Deep Brain Stimulation–Globus Palidus interna;* ***L-DOPA****: Levodopa Medication;* ***UPDRS-III****: United Parkinson's Disease Rating Scale, Part III;* ***N/A****: Not Applicable;* ***N/R****: Not Reported* | | | | | | | | | | | | | | | | |
|  |  |  |  |  |  |  |  |  |  |  |  |  |  |  |  |  |
|  |  |  |  |  |  |  |  |  |  |  |  |  |  |  |  |  |
|  |  |  |  |  |  |  |  |  |  |  |  |  |  |  |  |  |
