# Supplementary material for: Medical device-based neuromodulation for motor symptoms in Parkinson’s disease: a systematic review and meta-analysis
Source: Front Neurol. 2026 Feb 12;17:1731885. doi: 10.3389/fneur.2026.1731885 (PMC12937557; doi:10.3389/fneur.2026.1731885)

**Appendix 1.**

**Search Strategies:**

Pubmed

(("Parkinson Disease"[MeSH] OR "Parkinson's disease" OR "PD" OR "Parkinsonism")

AND

("Neuromodulation"[MeSH] OR "Deep Brain Stimulation"[MeSH] OR "Transcranial Magnetic Stimulation"[MeSH] OR "DBS" OR "TMS" OR "Vagus Nerve Stimulation"[MeSH] OR "tDCS" OR "Focused Ultrasound" OR "FUS" OR "Electroconvulsive Therapy" OR "ECT" OR "Spinal Cord Stimulation" OR "SCS"))

AND

(("Unified Parkinson Disease Rating Scale"[MeSH] OR "UPDRS" OR "UPDRS Part 3" OR "motor function" OR "motor symptoms" OR "bradykinesia" OR "tremor" OR "rigidity"))

Embase

('Parkinson disease'/exp OR 'Parkinson's disease' OR 'PD' OR 'Parkinsonism')

AND

('Neuromodulation'/exp OR 'Deep Brain Stimulation'/exp OR 'Transcranial Magnetic Stimulation'/exp OR 'DBS' OR 'TMS' OR 'Vagus Nerve Stimulation'/exp OR 'tDCS' OR 'Focused Ultrasound' OR 'FUS' OR 'Electroconvulsive Therapy' OR 'ECT' OR 'Spinal Cord Stimulation' OR 'SCS')

AND

('Unified Parkinson Disease Rating Scale'/exp OR 'UPDRS' OR 'UPDRS Part 3' OR 'motor function' OR 'motor symptoms' OR 'bradykinesia' OR 'tremor' OR 'rigidity')

Web of Science

TS=("Parkinson Disease" OR "Parkinson's disease" OR PD OR Parkinsonism)

AND

TS=("Neuromodulation" OR "Deep Brain Stimulation" OR DBS OR "Transcranial Magnetic Stimulation" OR TMS OR "Vagus Nerve Stimulation" OR "tDCS" OR "Transcranial Direct Current Stimulation" OR "Focused Ultrasound" OR FUS OR "Electroconvulsive Therapy" OR ECT OR "Spinal Cord Stimulation" OR SCS)

AND

TS=("Unified Parkinson Disease Rating Scale" OR UPDRS OR "UPDRS Part 3" OR "motor function" OR "motor symptoms" OR bradykinesia OR tremor OR rigidity)
AND

TS=("randomized controlled trial" OR "randomised controlled trial" OR "RCT" OR "randomized trial" OR "randomised trial" OR "controlled clinical trial" OR "double-blind" OR "single-blind" OR "placebo-controlled" OR "random allocation")

**Search date range:**

January 1^st^, 1960 (the concept of using electrical stimulation to influence neural activity dates back to the 1960s) to April 1st, 2025.

**Risk of bias assessment**

Risk of bias assessment for each included study across five RoB 2 domains.
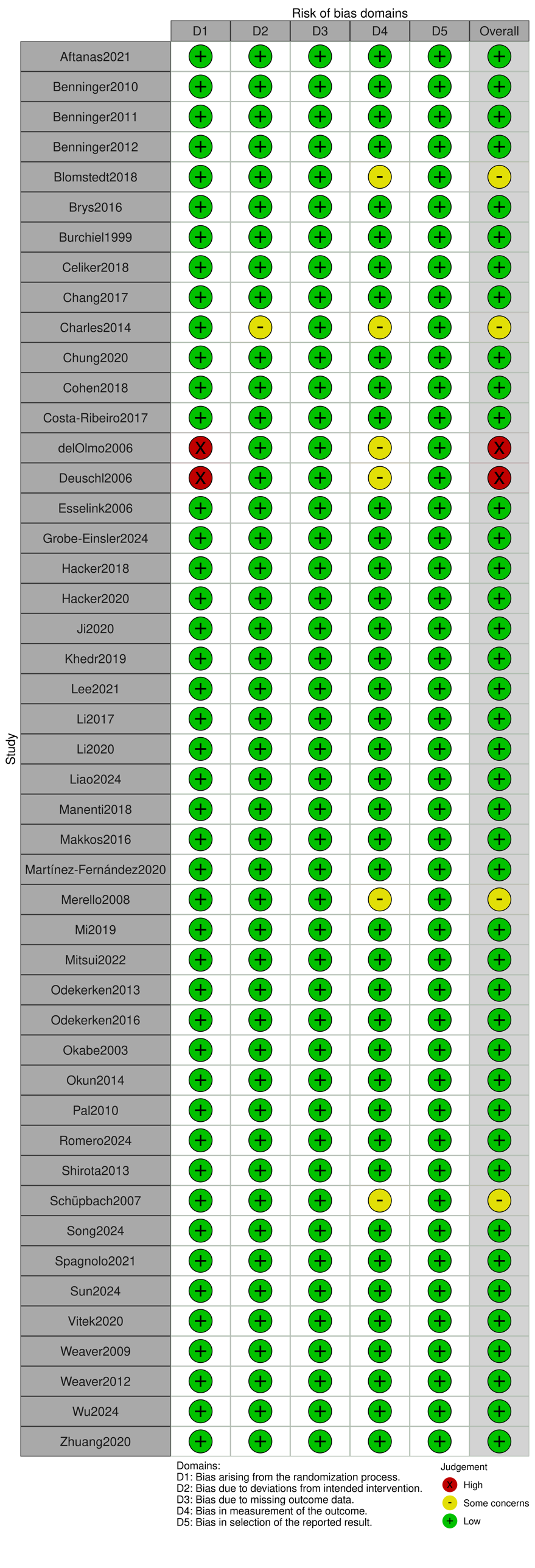


Proportion of studies rated as low risk (green), some concerns (yellow), or high risk (red) for each domain and for the overall risk of bias.


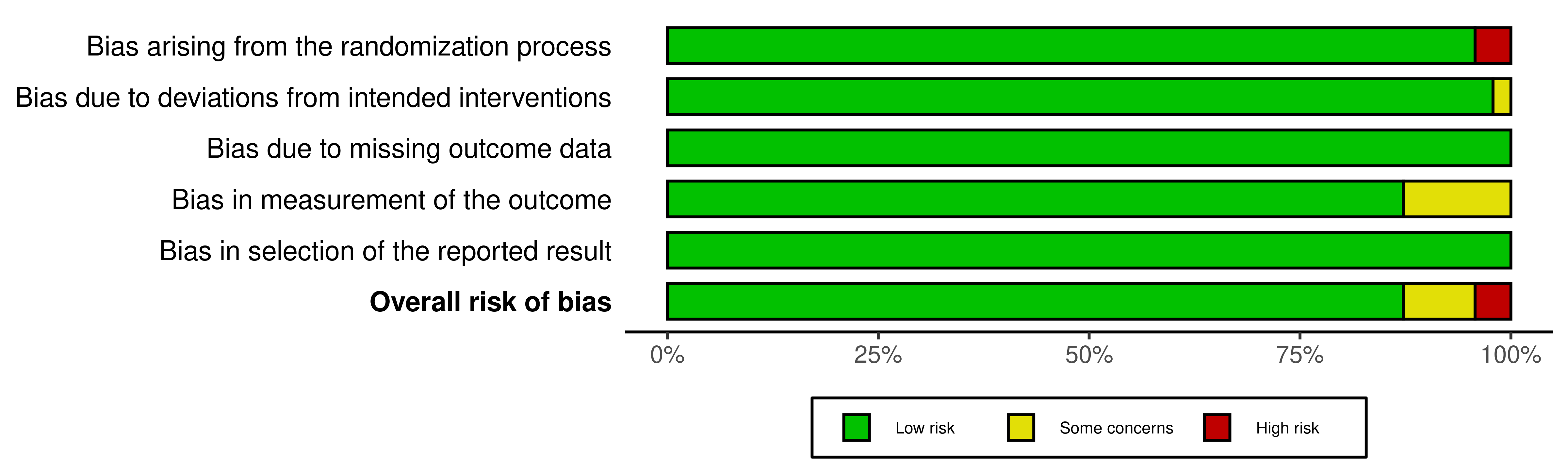


1. **Sensitivity Analysis Forest Plots**
2. Follow-Up Duration - Short, and Long-Term Follow-up.

Short Term

Long Term

1. Medication State at Outcome Assessment

ON State


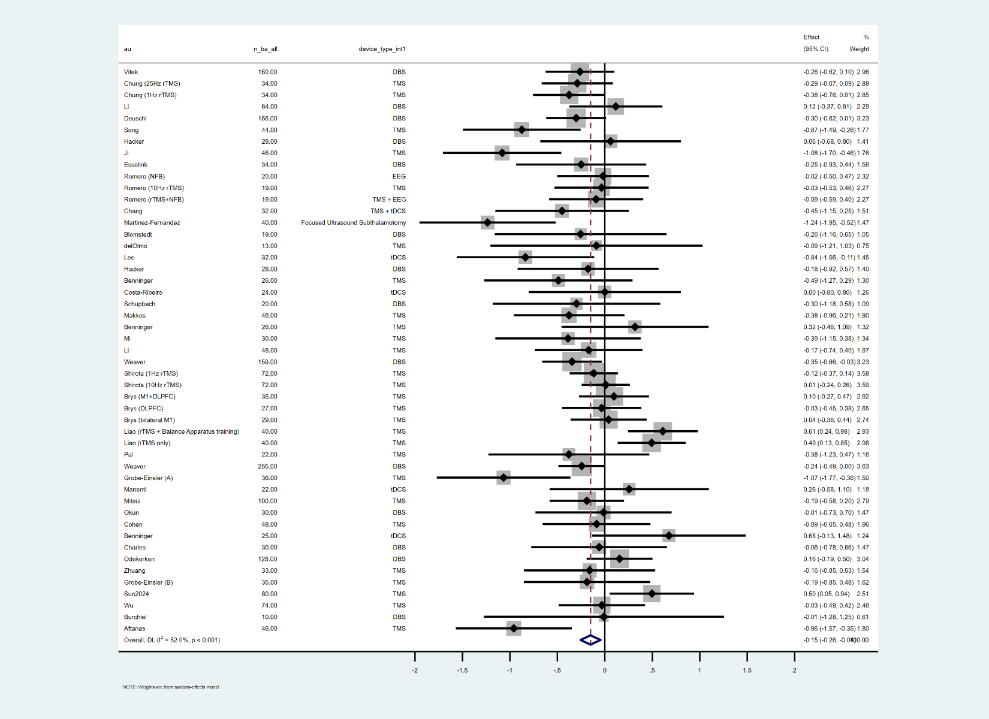


OFF State

1. Intervention Type

Invasive

Non-Invasive


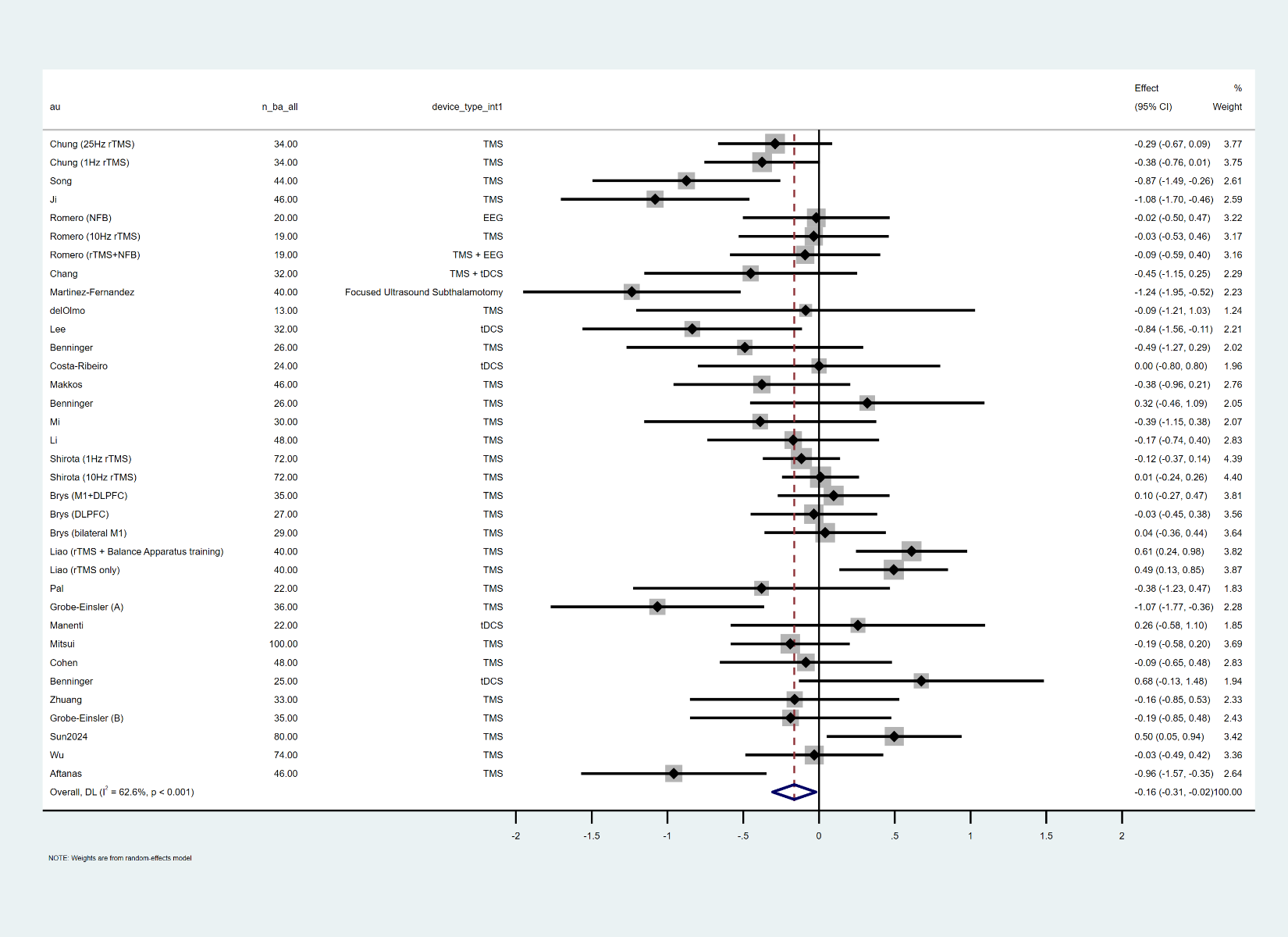


1. Outcome Measurement Scale

MDS-UPDRS3

UPDRS3

1. Outcome Measurement Scale

Stimulation Target

M1 (Primary Motor Cortex)


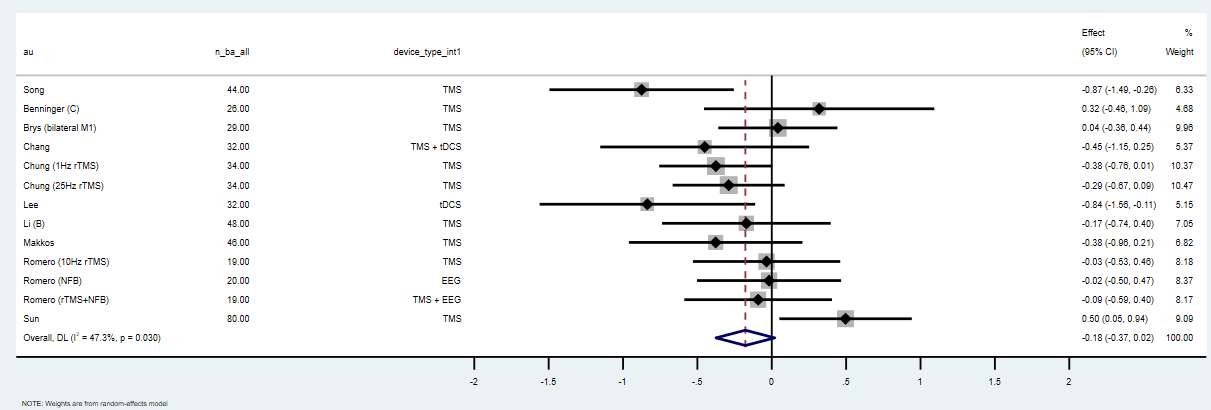


PFC (Pre Frontal Cortex)


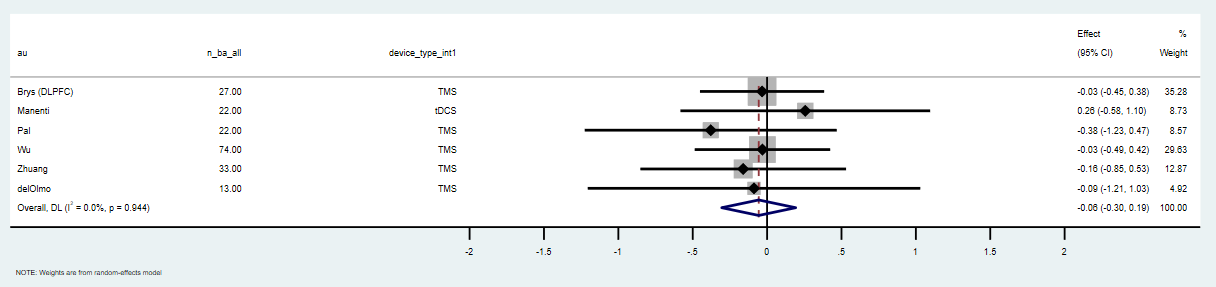


M1 + PFC


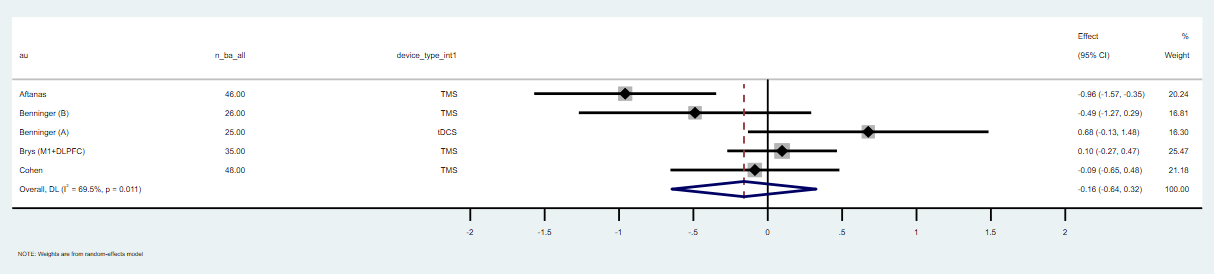


Supplementary Motor Area (SMA)


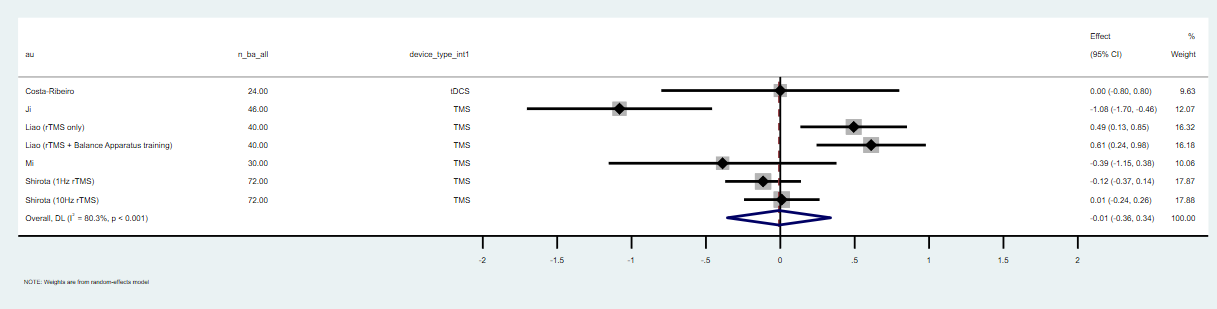


DBS – STN (Sub-Thalamic Nucleus)


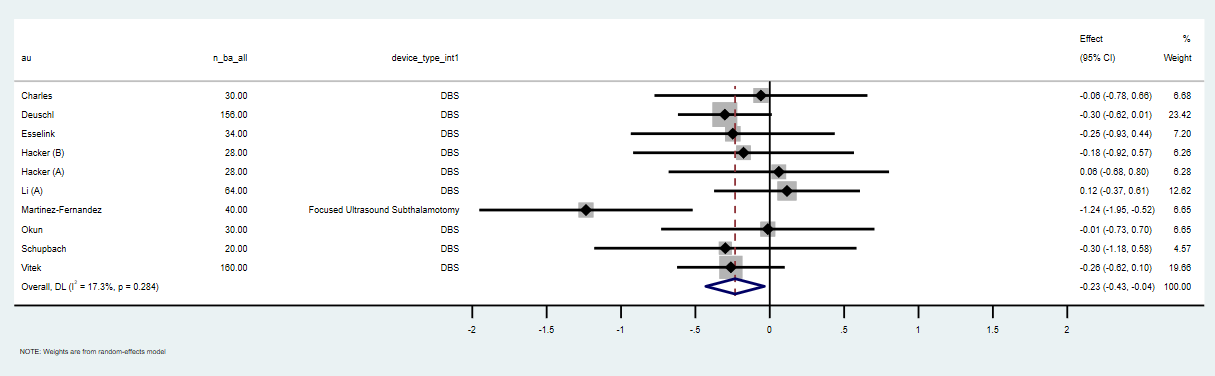


DBS – Gpi (Globus Palidus Interna)


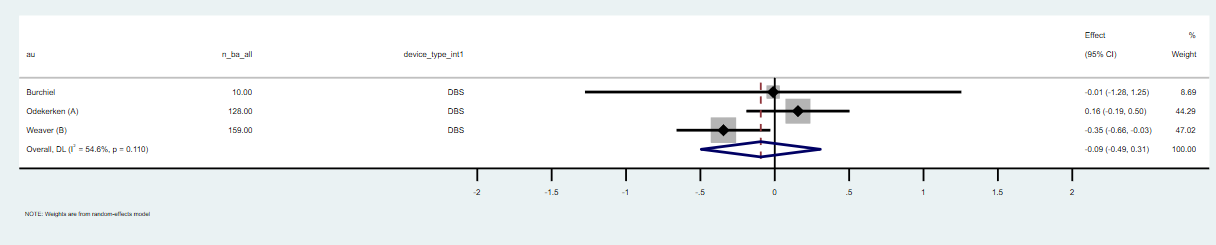


1. Outcome Measurement Scale

rTMS Stimulation Protocols

High Frequency (>5Hz)


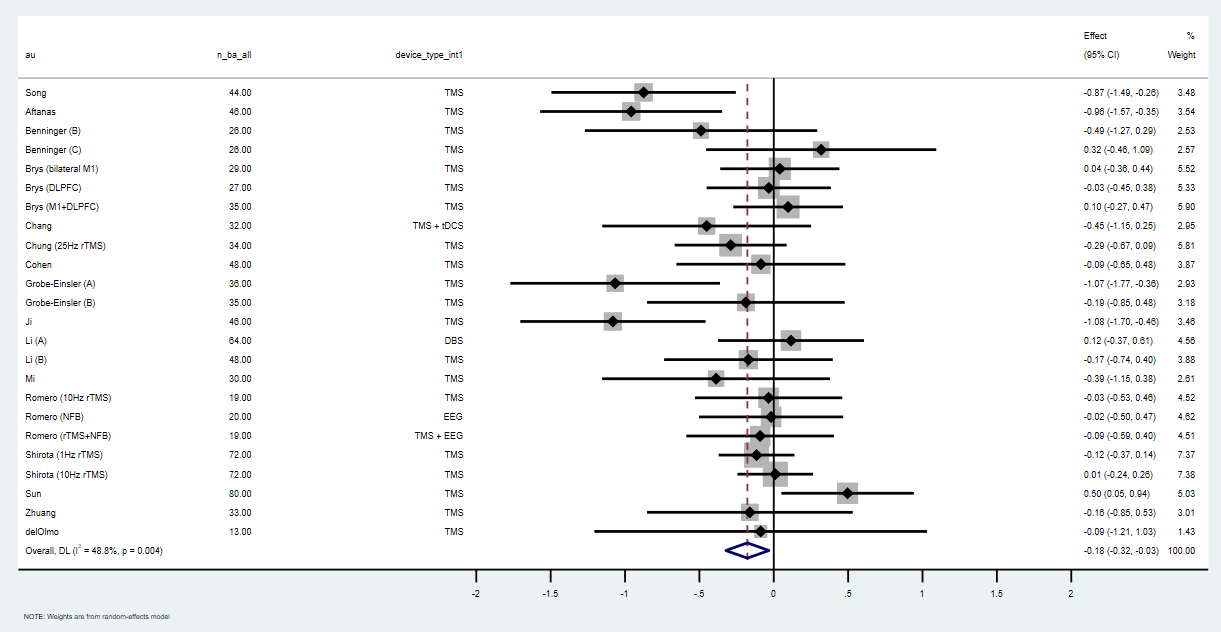


Low Frequency (≤ 5Hz)


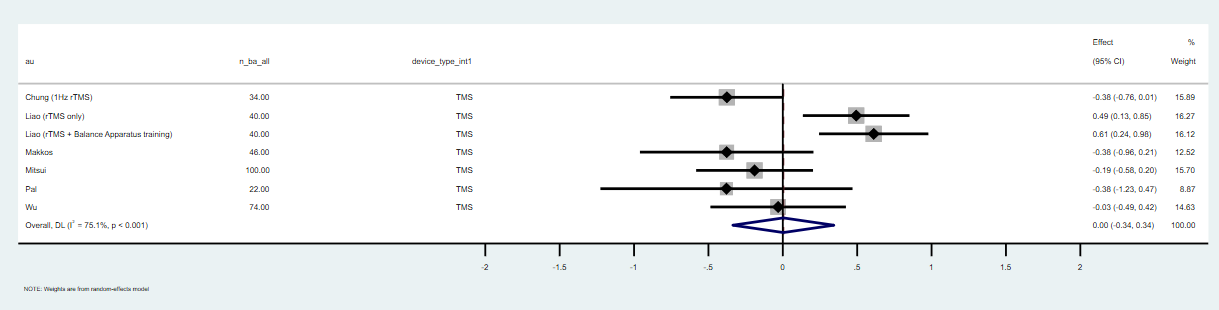

Supplement: Supplementary file 2 [file Data_Sheet_1.docx]
